# Preventing urinary tract infection in older people living in care homes: the ‘StOP UTI’ realist synthesis

**DOI:** 10.1136/bmjqs-2023-016967

**Published:** 2024-08-08

**Authors:** Jacqui Prieto, Jennie Wilson, Alison Tingle, Emily Cooper, Melanie Handley, Jo Rycroft Malone, Jennifer Bostock, Heather Loveday

**Affiliations:** 1 University of Southampton, Southampton, UK; 2 Richard Wells Research Centre, University of West London—Brentford Site, Brentford, UK; 3 UK Health Security Agency, London, UK; 4 University of Hertfordshire, Hatfield, UK; 5 Lancaster University, Lancaster, UK; 6 King's College London, London, UK

**Keywords:** leadership, nursing homes, adverse events, epidemiology and detection, infection control, safety culture

## Abstract

**Background:**

Urinary tract infection (UTI) is the most diagnosed infection in older people living in care homes.

**Objective:**

To identify interventions for recognising and preventing UTI in older people living in care homes in the UK and explain the mechanisms by which they work, for whom and under what circumstances.

**Methods:**

A realist synthesis of evidence was undertaken to develop programme theory underlying strategies to recognise and prevent UTI. A generic topic-based search of bibliographic databases was completed with further purposive searches to test and refine the programme theory in consultation with stakeholders.

**Results:**

56 articles were included in the review. Nine context–mechanism–outcome configurations were developed and arranged across three theory areas: (1) Strategies to support accurate recognition of UTI, (2) care strategies for residents to prevent UTI and (3) making best practice happen. Our programme theory explains how care staff can be enabled to recognise and prevent UTI when this is incorporated into care routines and activities that meet the fundamental care needs and preferences of residents. This is facilitated through active and visible leadership by care home managers and education that is contextualised to the work and role of care staff.

**Conclusions:**

Care home staff have a vital role in preventing and recognising UTI in care home residents.

Incorporating this into the fundamental care they provide can help them to adopt a proactive approach to preventing infection and avoiding unnecessary antibiotic use. This requires a context of care with a culture of personalisation and safety, promoted by commissioners, regulators and providers, where leadership and resources are committed to support preventative action by knowledgeable care staff.

**WHAT IS ALREADY KNOWN ON THIS TOPIC**

- Urinary tract infection (UTI) is common in older people living in care homes and drives the use of antimicrobial agents and hospital admissions.
- It can be harder to recognise in older people who often present with non-specific symptoms.

**WHAT THIS STUDY ADDS**

- Our realist synthesis highlights the importance of incorporating UTI recognition and prevention into the personalised fundamental care of residents in care homes as a proactive approach to preventing infection and avoiding unnecessary antibiotic use.

**HOW THIS STUDY MIGHT AFFECT RESEARCH, PRACTICE OR POLICY**

- By linking UTI recognition and prevention to fundamental care as a proactive and personalised approach to promoting the health and well-being of care home residents, our findings have the potential to transform practice.

## Introduction

 Urinary tract infection (UTI) accounts for the most antibiotic prescriptions in older people living in care homes in the UK^1^ and is a frequent cause of admission to hospital.^2^ Consequences of UTI can range from a mild self-limiting illness to severe sepsis with a mortality rate of 40%.^3^ Care home residents are four times more likely than older people living in their own homes to have a UTI caused by antibiotic-resistant bacteria.^4^ Older people who experience repeated episodes of UTI, and therefore frequent exposure to antibiotics, are at greater risk of acquiring resistant pathogens associated with bloodstream infections.^5 6^

Although most UTI in this setting are not associated with an invasive device, the presence of an indwelling urinary catheter (IUC) increases the risk of UTI by 3–8% per day.^7^ A prevalence survey of 425 care homes in the UK found that 6.9% of the 12 827 resident population had a urinary catheter.^8^ This study also provided evidence of variation in practice both in relation to discharge from the hospital with an IUC and its removal once in the care home suggesting there is room for a more proactive approach to reducing catheter use.

Guidance about strategies for UTI recognition and prevention in care homes is limited and does not account for the varying contexts in which care is delivered, the challenges presented by residents with complex health needs or the demands of care delivery by non-registered care staff with limited support from registered nurses.^9^,^11^ In addition, overdiagnosis of UTI is a problem as older people often present with non-specific symptoms which are difficult for care staff to interpret.^12^ Effective strategies to support both the accurate recognition and prevention of UTI in care home residents are important as they are interlinked approaches to reducing antimicrobial resistance.^13^ Accurate recognition is important to target and measure the effect of UTI prevention strategies and to reduce unnecessary antibiotic prescribing.

This review aimed to fill a gap in the international evidence base by creating an evidence-informed theoretical explanation of why interventions may or may not work in supporting both accurate recognition and prevention of UTI in older people in UK care homes. Our objectives were:

1. To identify which interventions could be effective, the mechanisms by which they work, for whom and under what circumstances.
2. To understand what needs to be in place in care homes for evidence-based interventions to be successful in reducing the harm from UTI.

### Research question

Preventing UTI among older people living in care homes: What works, for whom, in what circumstances and why?

## Methods

**Figure 1:**
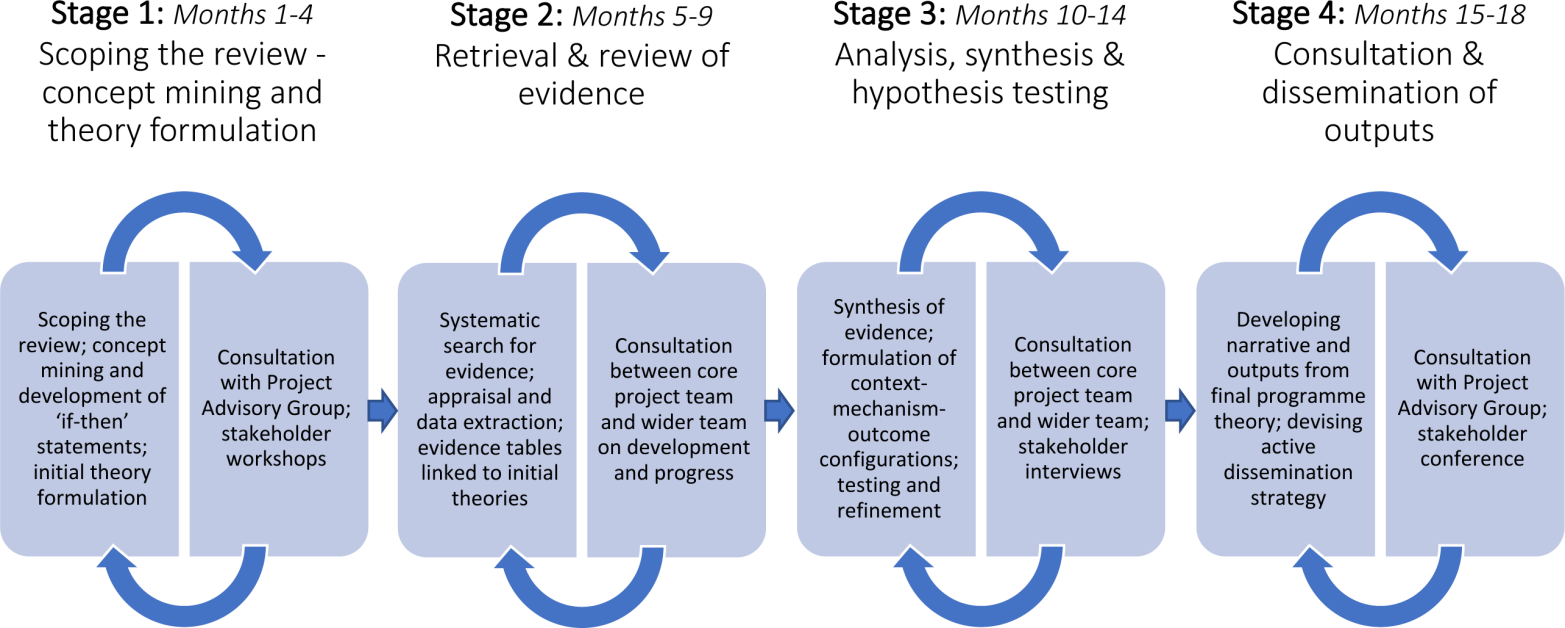
Stages of the review.

We undertook a realist synthesis in four stages over 18 months^14^ (figure 1) and followed RAMESES (Realist And Meta-narrative Evidence Syntheses: Evolving Standards) publication standards for reporting our findings.^15^

Stakeholder engagement was a key element of review stages 1, 3 and 4, informing the scope of the review, providing insights into the recognition and prevention of UTI in care homes and identifying how the theories we proposed would operate in the reality of practice. Initial discussions with our project advisory group helped to define and shape the scope of the review. In stage 1, we conducted four online theory-building workshops with a total of 36 stakeholders. Two of these workshops were with care home managers and their staff and two were with clinicians and specialist practitioners with a role in care homes. We had planned to include care home residents and family carers in these workshops but this was not possible in the context of the COVID-19 pandemic. However, we did interview a care home resident, a family carer and a lay representative. In stage 3, we undertook realist ‘teacher-learner’ interviews with practitioners (n=9) working in care homes. This is where theories are placed before participants who are invited to comment on their plausibility and whether or not they resonate with their experiences. In stage 4, we held an online stakeholder conference with representatives of care homes, primary care practitioners, commissioners, regulators and third-sector organisations to test the clarity and fit of our theories.

In September 2020, during stage 1 of the study, we conducted a topic-based scoping search of bibliographic databases to identify research literature on the prevention and recognition of UTI and catheter-associated UTI (CAUTI) in older people in long-term care facilities. Databases included Ovid MEDLINE, CINAHL Plus, Ovid EMBASE, Cochrane Library, Web of Science Core Collection, Sociological Abstracts via ProQuest, BiblioMaps and NIHR Journals Library. Sources also included review articles and highly cited index studies which were used to search for contemporaneous papers with a shared context using Google Scholar and ‘Publish or Perish’ software (see online supplemental file 1 for stage 1 search strategy).

Non-English language articles were excluded due to a lack of resources for translation. A 10-year date limit from January 2010 was applied to the stage 1 search taking account of both the relevance and volume of the literature retrieved to achieve a manageable approach without excluding important key studies. We considered this time period to encompass the more prominent policy focus on UTI and CAUTI in the UK care home sector as part of the imperative to prevent antimicrobial resistance and bloodstream infections caused by gram-negative organisms.

**Figure 2:**
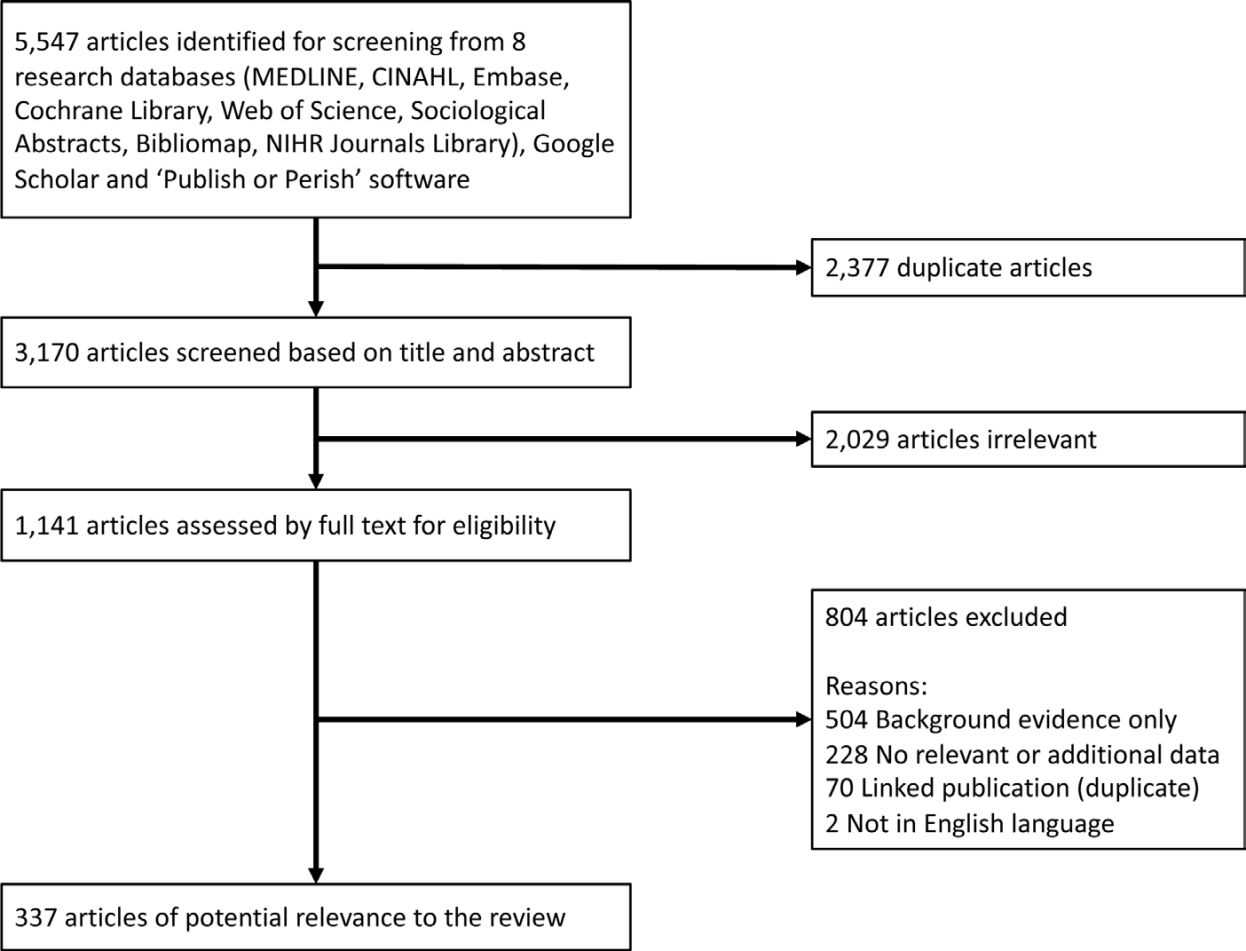
Flow diagram of the scoping search process in stage 1.

All articles were stored in EndNote and transferred to Covidence for de-duplication, screening for inclusion and relevance and rigour assessment. Two members of the research team independently undertook stage 1 initial inclusion screening using prespecified criteria (box 1). Disagreement was resolved through discussion or by a third reviewer. The results of the screening for the stage 1 searches are shown in figure 2.

Box 1 **Selection criteria**

**Population and setting**

- Older people (60 years plus) in care homes or other long-term care settings (not learning disability)

**Study design**

- Primary quantitative and qualitative studies such as intervention studies, surveys of knowledge/practice, observation of practice, interviews/focus groups, case studies
- Systematic reviews
- Guidelines, recommendations, policy
- Narrative review, commentaries, case reports, regulatory inquiries

**Related papers**

- Address other aspects of care or service delivery that influence or inform urinary tract infection prevention, for example, quality, safety, workforce, antimicrobial stewardship
- Epidemiological or antimicrobial resistance data that provide context and background to the topic

Concept mining^16^ was used to map evidence about approaches to recognising and preventing UTI, how they might work and reported enablers or barriers to implementation.

**Figure 3:**
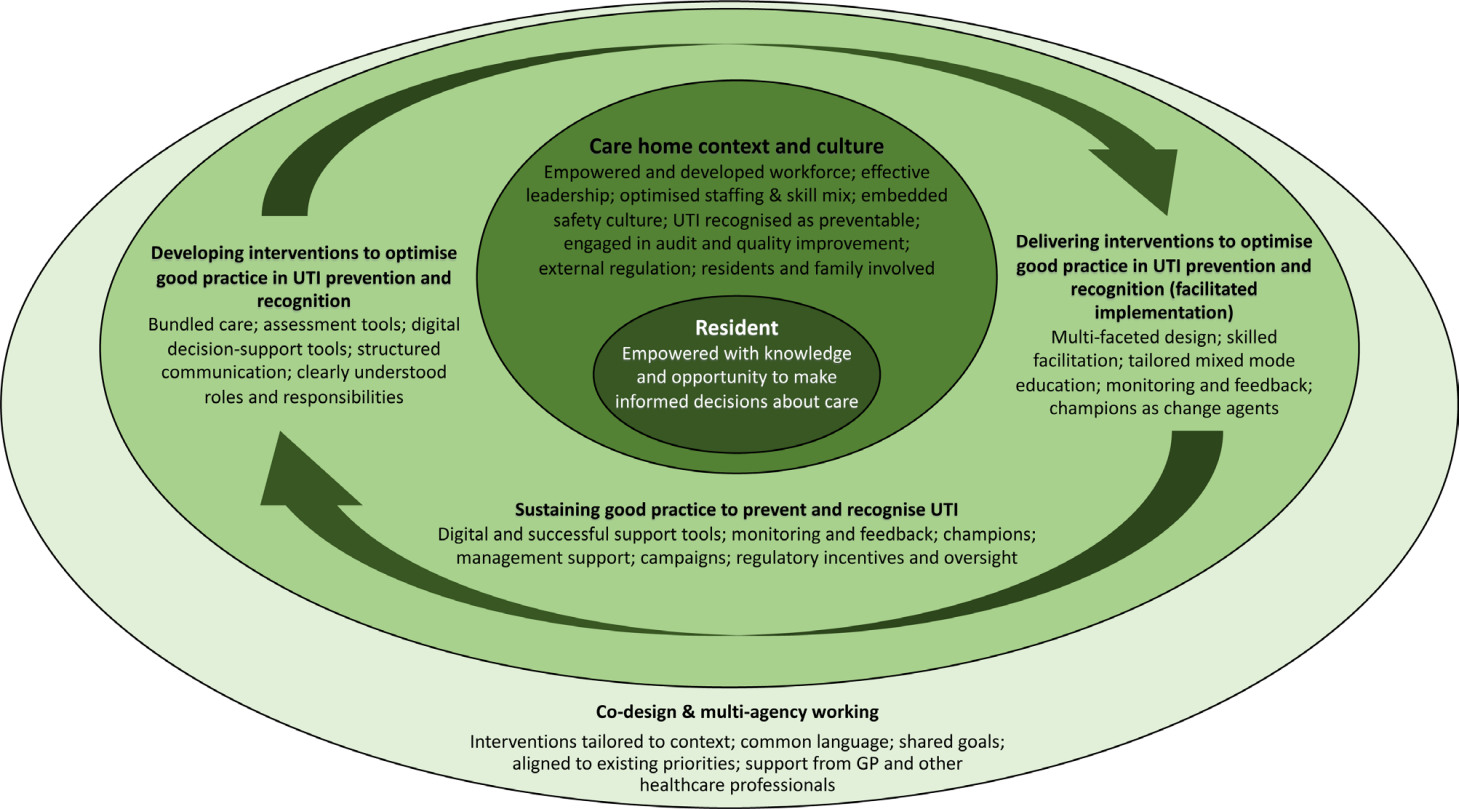
Conceptual diagram of initial programme theory. GP, general practitioner; UTI, urinary tract infection.

We established initial programme theory areas, expressed as a conceptual diagram (figure 3) and ‘if-then’ statements (online supplemental file 2) which made tentative links between interventions, outcomes and contexts within which care staff resources and responses (mechanisms) may operate. Preliminary hypotheses were developed by aligning the evidence on the effectiveness of interventions with context-dependent elements of implementation programmes in care homes. In stage 2, purposive searches for published and grey literature supporting these initial programme theory areas were undertaken (online supplemental file 3). The stage 2 supplemental searches were more targeted exclusively to inform the realist synthesis.

Two researchers used a ‘relevant and good enough’ flow chart (online supplemental file 4, figure 5) to complete the assessment of relevance (the extent to which evidence contributed to theory building, testing and refinement) and rigour (the extent to which the methods used to generate data were credible and trustworthy).^17 18^ The potential for a study to offer credible explanatory evidence despite weak methodological quality was also considered.^19^ Studies were excluded if the detail was insufficient to support refinement or testing of the programme theories.

Data were extracted using a bespoke data extraction form (online supplemental file 5, table 2) to populate theory areas with evidence on what appears to work, for whom, how and in what contexts.^20^ The data extraction process was shared among six team members with 50% of included studies being checked by a second researcher.

Context–mechanism–outcome configurations (CMOc) were developed through a process of analysis and synthesis of findings from included studies, their relation to the initial programme theories and requirements of effective interventions and with input from our project advisory group and stakeholders.^19^

## Results

2038 articles underwent full-text screening, of which 367 were identified as potentially relevant to the programme theories (online supplemental file 6, figure 6). Of these articles, 56 were included in the review (online supplemental file 7, table 3), 32 from the stage 1 scoping search and 24 added in stage 2.

The results of the scoping search and findings of the theory-building workshops with stakeholders in stage 1 were organised into four initial theory areas for further exploration:

1. Developing interventions to optimise good practice.
2. Delivering and sustaining good practice.
3. Care home context and culture.
4. Co-design and multi-agency working.

Our deliberations led us to propose that care home context and culture including the resources available and the perspective taken on safety and quality were central to the design, delivery and sustainability of interventions described in the literature we had identified. Figure 3 illustrates the relationship between these elements.

The scoping search found insufficient evidence to develop a theory around staffing levels and UTI specifically, although this was reflected in the wider evidence on care home context and culture. From the stage 2 supplemental searches, there was insufficient evidence to develop a stand-alone theory about family involvement but this formed a thread through other theories. Although UTI could be a contributory factor to urinary incontinence, there was a lack of evidence that urinary incontinence was a cause of UTI or that incontinence pads affected the risk of UTI.

**Figure 4:**
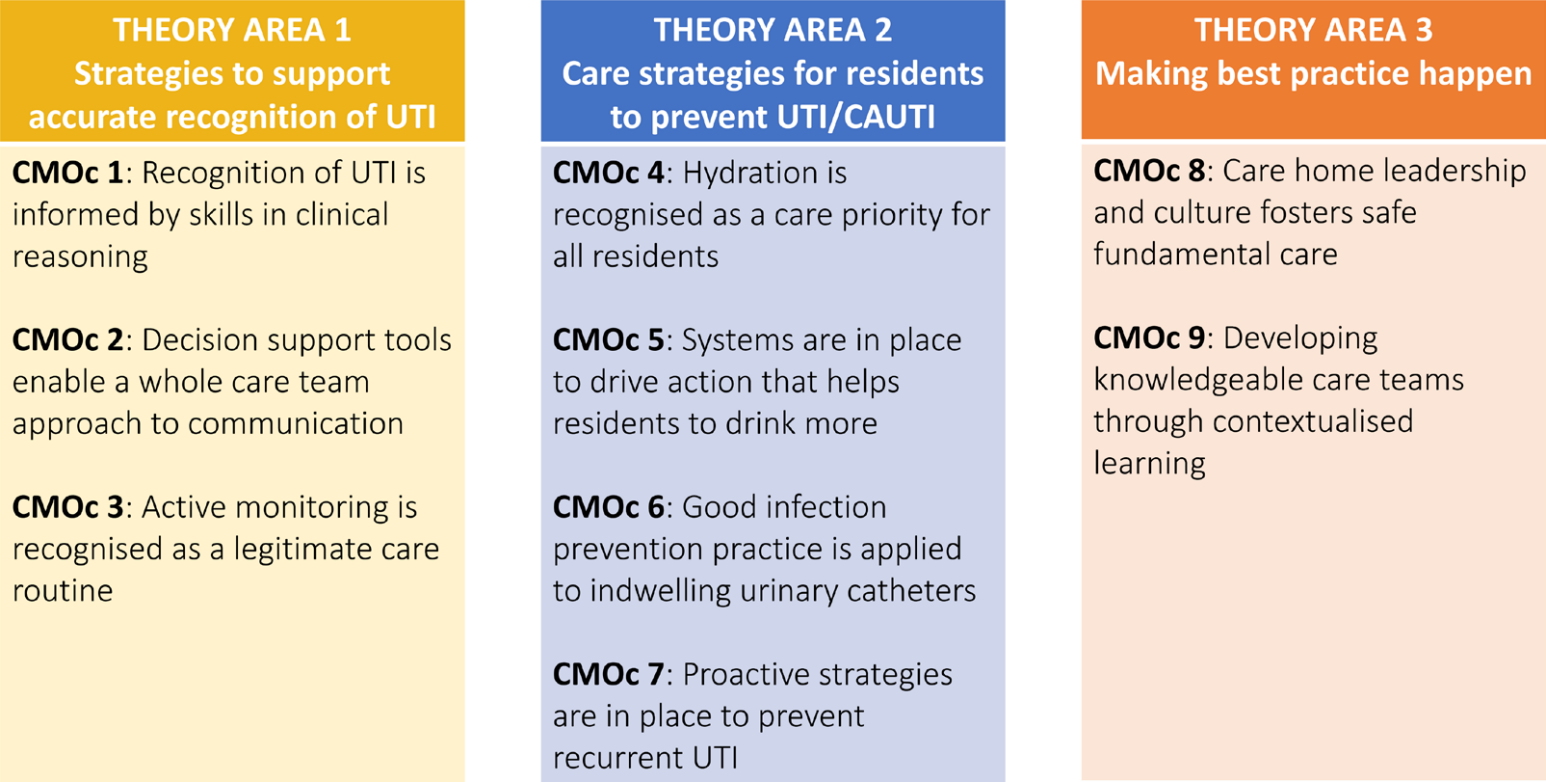
Theory areas and related context–mechanism–outcome configurations (CMOc). CAUTI, catheter-associated UTI; UTI, urinary tract infection.

Evidence included in the review informed the development of nine CMOc which were arranged into three theory areas (figure 4). Theory areas 1 and 2 comprise strategies to recognise and prevent UTI respectively whereas theory area 3 encompasses cross-cutting concepts of leadership and workforce development that are of relevance to them both. A summary of each CMOc is presented in online supplemental file 8, table 4.

### Theory area 1: strategies to support accurate recognition of UTI

#### CMOc 1: recognition of UTI is informed by skills in clinical reasoning (n=11 articles)

In care homes where there is a commitment to supporting shared learning (context), educational interventions on UTI that are tailored to the role and work of care staff (context) can enable them to develop the knowledge and confidence to consider alternative explanations for changes in a resident’s condition (mechanism) reducing the likelihood of UTI being the default explanation (outcome).

Stakeholders highlighted the importance of accurate recognition of UTI and the need for care home staff to know how to distinguish ‘soft signs’ of UTI from another condition. The evidence demonstrated it can be difficult for care home staff to change their intuitive understanding of UTI.

Of the 11 articles that addressed this CMOc,^21^,^31^ five were reports of three intervention studies^21 22 25 28 29^ designed to reduce inappropriate prescribing for UTI in nursing homes through tailored education and the use of assessment and communication tools combining technical evidence-based content with experiential knowledge. Two of these studies^25 28^ found that drawing on participants’ experience and understanding of UTI challenged their thinking, improved their clinical reasoning and enabled them to consider alternative explanations for generalised changes in a resident. Application of learning to practice was important to embed learning and implement the intervention.^25 28 29^ This was facilitated by managers and senior care staff through both formal and informal opportunities to review residents with soft signs and by regular visits to care environments to raise awareness of the intervention.^25 28^

#### CMOc 2: decision support tools enable a whole care team approach to communication (n=10 articles)

Decision support tools that are codesigned by care home staff and involve the whole care team in the recognition and prevention of UTI (context) can enable and motivate junior staff to communicate their observations and concerns using a shared language (mechanism), supporting the accurate diagnosis of UTI and appropriate antibiotic prescribing (outcome).

Stakeholders highlighted the importance of care home staff being able to recognise a UTI and communicate this to colleagues and general practitioners. The evidence demonstrated the need to involve the whole care team in the process of gathering and conveying information about a resident’s signs and symptoms with tools that use a shared language to support assessment and decision-making.

Of the 10 articles that addressed this CMOc,^2122 25 29^,^35^ five were reports of four intervention studies^21 22 25 29 33^ that tested structured assessment and communication tools, two of which^21 25^ were developed with care staff to align with their working patterns and observed presentations. The perceived usefulness of assessment tools was influenced by the degree of fit to the resident’s symptoms. When there was good alignment, these decision support tools provided a reference for symptoms of infection and actions to take but they were less useful when presenting symptoms were not listed.^21 25^

Decision support tools were perceived to provide care staff with a shared vocabulary and structure which helped them to communicate logically and with increased confidence. The evidence highlighted the importance of clarifying roles within teams^25 33^ and recognising the value of observations made by junior staff who work most closely with residents and who may lack confidence or experience hierarchical barriers to conveying their observations.^25 30 31^

#### CMOc 3: active monitoring is recognised as a legitimate care routine (n=17 articles)

Use of an active monitoring protocol which is accepted by the care team and family carers as a proactive step in response to diagnostic uncertainty about UTI (context), can empower care staff to engage in active monitoring and support shared decision-making (mechanism), increasing the likelihood of a correct UTI diagnosis and reducing the potential for overuse of antibiotics (outcome).

Active monitoring is a period of increased monitoring when a resident’s signs and symptoms do not yet suggest a UTI specifically. Stakeholders acknowledged active monitoring of residents with non-specific symptoms of UTI was important but identified that it could be challenging where residents experienced frequent UTI as their family might be reluctant to delay treatment when there was uncertainty about a diagnosis.

The evidence described how general practitioners rely on the observations and actions of care home staff to inform their decision-making and how they can experience pressure from staff and family to prescribe antibiotics, even if diagnostic criteria are unclear.^36 37^

17 articles addressed this CMOc^21^,^2729 36^ of which four^22 29 41 42^ reported intervention studies that showed how active monitoring as part of a decision support tool or process can increase its use and decrease antibiotic use without adversely affecting UTI-related hospital admissions or other harms. Active monitoring periods within a well-defined UTI management protocol offer care staff the opportunity to reflect before deciding how to respond, to check for other underlying causes of changes before contacting a clinician^21 23^ and discourage fast, intuitive decision-making. There was evidence that decision support tools incorporating active monitoring helped care staff discuss the management of infection with family carers and written information was important to increase relatives’ awareness of the approach.^25^

### Theory area 2: care strategies for residents to prevent UTI/CAUTI

#### CMOc 4: hydration is recognised as a care priority for all residents (n=7 articles)

Prioritisation of hydration as a core activity by managers with the provision of resources that meet residents’ needs and preferences (context) can enable care staff to support residents to drink regularly throughout the day (mechanism) and reduce their risk of dehydration and UTI (outcome).

Stakeholders identified some important barriers to ensuring frail older people residing in care homes consume enough fluids including lack of education, staff time and inadequate choice of fluids and equipment to meet residents’ preferences.

Seven reports of five studies described multimodal interventions aimed at increasing hydration and preventing UTI in care home residents.^45^,^51^ All were pragmatic in design reflecting the challenges of conducting research in a care home setting.^45^,^50^ Care routines were changed to ensure that hydration was prioritised by staff and initiatives involved the whole care team. Increasing the number of drinking opportunities, both opportunistically and as part of mealtimes or other organised activities, and extending the choice of drinks ensured resources of staff, equipment and fluids were available to support and assist residents to drink. The impact of interventions was observed by increased fluid intake, reduction in laxative and antibiotic consumption and reduction in UTI and other adverse health events including falls and hospital admissions.^47 49 50^

Staff education as part of a multimodal approach increased understanding of the importance of hydration in reducing the risk of UTI, challenged beliefs about ‘appropriate’ fluids and helped staff to recognise how normal patterns of care can limit residents’ fluid consumption.^4547 49^,^51^ However, training is unlikely to be effective if care staff are not empowered to initiate system changes and influence their colleagues to focus on hydration.^45^

Care home managers were key to prioritising the time available to staff to undertake drinks rounds and support residents to drink. Care homes with strong leadership and management support were more likely to demonstrate success by endorsing hydration as a critical care activity and ensuring it was integrated into care routines.^46 47 49 50^

#### CMOc 5: systems are in place to drive action that helps residents to drink more (n=5 articles)

Systems that accurately measure whether residents are meeting their individual target daily fluid intake (context), can motivate staff to support the resident to achieve the target (mechanism) and take timely corrective action when a residents’ fluid intake is poor (outcome).

Knowing how much fluid a resident has consumed is important because it ensures that poor intake is noticed and addressed by staff. However, documentation and monitoring of all residents’ fluid consumption is often inaccurate.^47 49^ Defined systems or processes for both accurately monitoring intakes and acting in response to residents with poor fluid consumption can be facilitated by approaches such as drinks dairies as an alternative to traditional fluid balance charts.^50^

In care homes where hydration is prioritised and supported by managers and nurse leaders, recording fluid consumption can drive action to prevent dehydration. In two studies, individual targets were calculated for each resident or those identified as at risk of dehydration based on a standardised formula.^49 51^ Fluid intake targets can create a tension between ensuring these targets are met while not forcing residents to drink and achieving optimal intakes for all residents can be difficult.^46 49^

Where managers, senior staff and/or nominated champions regularly reviewed data on drinks rounds and provided praise and positive feedback, hydration was signalled as a priority and staff were motivated to sustain the activity.^50 51^ Expert support from professional staff external to the home such as speech therapists, occupational therapists and dieticians and from care commissioners was also identified as a key success factor.^50 51^

#### CMOc 6: good infection prevention practice is applied to IUCs (n=5 articles)

In care homes where the benefit of minimising IUC use and implementing CAUTI prevention strategies is recognised and where residents and families are involved in decision-making (context), care staff can develop the confidence to apply the principles of infection prevention to the management of catheters and initiate their removal (mechanism), reducing the risk of CAUTI and use of antimicrobials (outcome).

Stakeholders identified that the number of residents with an IUC is relatively low although some residents were admitted from the hospital with an IUC without information about the reason for its insertion. This was also supported by the evidence we found.^8^

Three systematic reviews relevant to care home settings were identified.^52^,^54^ These provided evidence that CAUTI can be reduced by applying multimodal interventions that include a ‘trial without catheter’ for residents admitted with an IUC without appropriate indication, using protocols to avoid IUC for managing urinary retention and incontinence, discussing alternatives to IUC with residents, families and care staff and increasing staff knowledge about aseptic insertion and appropriate care.^52^ Interventions to develop staff confidence and empower them to initiate the removal of inappropriate IUC included evidence-based guidelines, education, improved communication at transfers of care, information for residents and family carers about the disadvantages of IUC and specific guidance to promote alternatives.^52 53^

A multimodal programme implemented in nursing homes in the USA was associated with a significant decrease in the incidence of CAUTI. The intervention comprised a technical bundle focusing on education and a behavioural bundle to develop leadership behaviours, resident and family engagement and effective communication. Improvement in catheter management was identified as the primary driver of CAUTI prevention rather than decreasing catheter days.^55^ Support from a specialist team, a range of educational tools and data capture and feedback systems were key to the success of this intervention.^56^

#### CMOc 7: proactive strategies are in place to prevent recurrent UTI (n=7 articles)

In care homes where recurrent UTI (RUTI) is recognised as a treatable problem with systems to identify residents at risk (context), care staff are less accepting of the inevitability of RUTI and can consider options for its prevention (mechanism), ensuring residents are offered preventative treatment to reduce the risk of further UTI and the emergence of antimicrobial resistant infections (outcome).

Despite the treatment of RUTI being accepted practice,^57 58^ it is not commonly used in care home residents. Awareness among care staff of pharmacological strategies for preventing UTI is limited, although hydration and cranberry juice are perceived as important.^26 27^ There are also concerns that labelling residents as having RUTIs would encourage unnecessary antibiotic treatment.^24^

The options for managing people with RUTI are complex and require evaluation of individual underlying risk factors to select the best approach. Stakeholders identified that systems to recognise residents with RUTI and trigger an assessment or treatment were deficient and staff were not aware of the availability and value of prophylactic treatment.

This CMOc was informed by national and international guidelines^57 58^ and one comprehensive narrative review.^59^ We found only one small study^60^ undertaken in a care home setting where a continence advisor developed individualised treatment plans for residents with RUTI with preventative strategies including topical oestrogen and increased fluid and fibre intake. The intervention was associated with an 80% reduction (31–6) in UTI highlighting the value of input from a continence advisor in preventing UTI.

### Theory area 3: making best practice happen

#### CMOc 8: care home leadership and culture fosters safe fundamental care (n=24 articles)

When care home managers and leaders consistently endorse and support resident-centred approaches to preventative fundamental care (context), their staff can prioritise and see the value of activities that support UTI recognition and prevention (mechanism) and sustain best practice (outcome).

Leadership and culture within care homes were referred to indirectly by stakeholders who considered organisational structures inclusive of all the care home team were important. The evidence we reviewed underlined the pivotal role of care home managers in the prioritisation and delivery of best practice relating to UTI recognition and prevention within care homes.^25 28 46 56^ This was supported in the wider literature on implementing changes in care homes.^61^,^63^

Evidence from the included studies identified stable care home leadership as central to optimal implementation with efforts easily undermined by management changes or constant staff turnover.^4647 55 56 61^,^65^ Managers demonstrated commitment through visible actions supporting an intervention and endorsement of changes necessary for implementation.^2533 61^,^63^ Developing policies and processes to reinforce best practice ensured that interventions remained a priority^46 55 56^ and gave staff the confidence and authority to incorporate changes in their work.^25 28 33 46 56 61 62^

Enabling staff to commit time to fundamental care was a key feature^46 47 56 61^ and alignment between the intervention and professional values of care staff was an important motivating factor.^28^ Staff empowerment and recognition of the benefits of changes were linked to increased job satisfaction.^37 56 61^

#### CMOc 9: developing knowledgeable care teams through contextualised learning (n=10 articles)

When education is contextualised to the roles of care staff (context), they can see the relevance of new learning about UTI and feel motivated (mechanism) to apply best practice to the care of residents (outcome).

There was a widespread recognition among stakeholders that education of care staff was crucial to enabling them to accept and participate in change, deliver high-quality care and feel confident in the information they communicated to colleagues and general practitioners. Education was a key component of many of the interventions in care homes and was important in changing perceptions about UTI and encouraging staff to recognise that a different approach was necessary.^21 28 33 47^

The most effective education for care home staff was contextualised so that it resonated with their experience and they could see the relevance to their practice.^17 25 28 45^ Using experiential learning or interactive approaches generated greater motivation and interest and helped participants to reflect more critically on the delivery of care and their role in improving it.^21^ The evidence supported socio-adaptive educational approaches with flexible materials, a variety of delivery modes and sufficient sessions^25 55 56^ to account for the learning needs of a multiskilled, multicultural workforce.^45 55^

Translating education into a change in practice also requires resources at the point of care to remind staff what they have learnt, for example, pocket cards and infographics.^55^ Short informal briefings or explanation, for example, huddles were found to be of value in supporting the whole team to adopt change.^25 45 50^ However, it was suggested that these may lose impact without case-based exercises that are more likely to encourage reflection.^28^ Supported hands-on practice, supervision and access to experts to model practice were recognised as helpful in developing skills and confidence.^28 66^ Delivering education in ways that facilitated ‘unlearning’ was found to be necessary to address pre-existing beliefs and assumptions that may adversely affect the care provided to residents and the implementation of an intervention to improve practice.^21 45^

## Discussion

This realist synthesis has developed programme theory to explain what works, for whom and under what circumstances for the recognition and prevention of UTI in older people living in care homes in the UK. It identifies what appears to be needed in care homes for evidence-based interventions to be successful in reducing the harm from UTI in this high-risk population. Our theory elucidates the vital role of care staff in recognising and preventing UTI and how this is enabled through care home leadership and workforce development that prioritises personalised fundamental care of residents.

Linking recognition and prevention of UTI to fundamental care^67^ can help care staff to recognise the importance of their role and that acquiring a UTI should not be inevitable for most residents. It enables care home managers to align prevention strategies to their wider organisational goals and regulatory requirements in supporting a culture of safety and personalised care. For example, understanding how supporting residents to drink sufficient fluids helps to reduce drowsiness, falls, confusion and UTI can empower staff to prioritise hydration care into their practices and care routines when this is actively endorsed by managers and leaders. Understanding and targeting personal barriers to drinking, such as fears about incontinence, enables personalised care.

Similarly, aligning UTI recognition with antimicrobial stewardship and the UK Government’s national action plan for antimicrobial resistance^13^ may help care staff to appreciate the extent to which inappropriate treatment of UTI makes infection more difficult to treat and impacts on resident health and well-being. Instilling a culture within care homes where generalised changes in a resident’s condition such as confusion and drowsiness lead to a holistic, evidence-based assessment rather than a presumption of UTI can help care staff to develop the confidence to consider alternative explanations for non-specific symptoms. Knowing the resident and working in partnership with family carers helps recognise the changes that might indicate UTI.

The theory we developed aligns with the principles of the National Health Service England enhanced health in care homes (EHCH) framework,^68^ a key Ageing Well programme set out in the National Health Service (NHS).^69 69^ The EHCH framework promotes proactive care delivery through a person-centred, whole system, collaborative approach.^68^ This move away from traditional reactive models of care delivery exemplifies how prevention is central to the health and well-being of care home residents. It enables emphasis on the role of care staff in preventing UTI as well as supporting its diagnosis and treatment.

In relation to the management of health and well-being, the EHCH framework identifies how recognising early signs of deterioration is important when a resident may be becoming unwell, to prevent any further deterioration and avoid escalation of care where possible.^68^ However, tools used in care homes to identify and escalate deterioration are not designed to facilitate active monitoring. Our realist synthesis suggests active monitoring could enable care staff to work in partnership with family carers and healthcare professionals when there is uncertainty about UTI to initiate preventative care, such as increasing fluid consumption and extra monitoring, ahead of escalation to reduce the inappropriate use of antimicrobials. This requires a culture within care homes where staff are supported to share their knowledge and observations and where they have clarity about their roles and responsibilities.

The evidence we found underlines the importance of ensuring care staff have access to educational activities that build knowledge, skills and confidence in the prevention and recognition of UTI. Education that is contextualised to the work and role of care staff uses and draws on their experience, challenging their assumptions and embedding new learning that enables and motivates them to question low-value practices and deliver best evidence-informed care.

Active and visible leadership by care home managers is essential to ensure the whole care team including residents, their families, care home staff and visiting health professionals are involved in incorporating best practice into daily care routines and to drive the prevention of UTI. The evidence we found suggests that stable care home leadership, staff resources to deliver fundamental care, some autonomy over implementing change and interventions that fit with daily work patterns are key to engaging staff and underpin the success of change efforts. These efforts can be easily undermined by management changes or constant staff turnover.^5556 61^,^65^ Sustained change is more likely when there are demonstrable benefits to residents and staff with access to resources and expertise, for example, continence advisors and infection prevention practitioners, to facilitate improvement. Priorities identified by regulators and commissioners of care influence what care home managers understand as important and how care home resources are deployed.

### Recommendations for practice

Actionable recommendations at both an organisational and system level are required to support UTI recognition and prevention in the care of older people living in care homes. Our recommendations are outlined in online supplemental file 9, table 5.

### Strengths and limitations

To our knowledge, this is the first realist synthesis to address the topic of UTI prevention and recognition in older people living in care homes. Using realist methodology enabled a rigorous approach, bringing together multiple types of evidence and a broad range of stakeholders to inform our theory-driven explanation of how interventions to improve the prevention and recognition of UTI might work in care homes for older people. This was important to establish the feasibility and usefulness of interventions in care homes for older people given the limited evidence from quantitative research studies and the contextual factors and variations between care home settings (eg, nursing provision, resident characteristics, access to support from community healthcare professionals) which influence how interventions can be implemented. Previous systematic reviews of evidence to reduce UTI in care home residents^52 70^ were unable to draw conclusions about the most effective interventions due to limited available evidence, heterogeneity of interventions and outcome measures and methodological limitations.

Our scoping review identified an extensive literature on UTI which focused mainly on recognition, prevention, diagnosis and treatment, antimicrobial resistance, antimicrobial stewardship interventions or epidemiology. The concepts of UTI recognition and prevention were rarely integrated with studies generally addressing either one or the other. The evidence we synthesised has highlighted synergies between the recognition and prevention of UTI which emphasise the value of integrating them into the design and delivery of person-centred care.

We were unable to include non-English language articles in our review as we had no resources for translation which increases the risk of publication bias. Studies focusing on the prevention of UTI and CAUTI in care home settings were predominantly from the USA and Europe where the regulatory and funding systems for long-term care of the elderly differ. For example, in the USA national reporting of UTI rates plays a significant role in driving system-wide improvements in care.^71^ Studies undertaken in the UK and Europe were focused primarily on interventions to reduce antimicrobial resistance through stewardship but had significant learning that was transferable to the prevention and recognition of UTI. Our synthesis tried to take account of these differences, but we are aware that we may not have reflected all realities.

## Conclusion

This realist synthesis addresses a gap in evidence by providing explanations for why interventions to improve the recognition and prevention of UTI in older people living in care homes may or may not work. By linking UTI recognition and prevention to fundamental care as a proactive and personalised approach to promoting the health and well-being of care home residents, our findings have the potential to transform practice. Ensuring care activities are integrated and prioritised within care home routines and systems for care delivery and are enabled through care home leadership and workforce development can help care staff to realise their vital role in UTI recognition and prevention.

## supplementary material

10.1136/bmjqs-2023-016967 online supplemental file 1

10.1136/bmjqs-2023-016967 online supplemental file 2

10.1136/bmjqs-2023-016967 online supplemental file 3

10.1136/bmjqs-2023-016967 online supplemental file 4

10.1136/bmjqs-2023-016967 online supplemental file 5

10.1136/bmjqs-2023-016967 online supplemental file 6

10.1136/bmjqs-2023-016967 online supplemental file 7

10.1136/bmjqs-2023-016967 online supplemental file 8

10.1136/bmjqs-2023-016967 online supplemental file 9

## Data Availability

All data relevant to the study are included in the article or uploaded as supplementary information.
