# Supplementary material for: Preventing urinary tract infection in older people living in care homes: the ‘StOP UTI’ realist synthesis
Source: BMJ Qual Saf. 2024 Aug 8;34(3):e016967. doi: 10.1136/bmjqs-2023-016967 (PMC11874410; doi:10.1136/bmjqs-2023-016967)
Supplement: online supplemental file 1 [file bmjqs-34-3-s001.pdf]

Supplementary File 1: Search strategy and summary of searches in Stage 1

OVERVIEW OF DATABASES AND NUMBERS OF RESULTS

Database	Date run	Results	Notes
CINAHL Plus with full text (EBSCOhost)	26/09/2020	619	
OVID MEDLINE	26/09/2020	669	
OVID Embase	26/09/2020	1870	
Cochrane Library	26/09/2020	295	5 Cochrane Reviews<br>290 trials
ASSIA	n/a	n/a	Not available at Southampton
Bibliomap	26/09/2020	0	
Sociological Abstracts (ProQuest)	01/10/2020	4	
Scopus	n/a	n/a	Used Web of Science instead
Web of Science – Core Collection	01/10/2020	865	Core Collection includes the Social Sciences Citation Index.
NIHR Journals Library	01/10/2020	2	StOP UTI and PRINCESS. The PRINCESS papers are already retrieved from the other databases.
TOTAL		4322	
TOTAL after deduplication		2648	

Review articles and highly cited index studies, were also used to search for contemporaneous papers with a shared context using Google Scholar and 'Publish or Perish' software.

MAIN SEARCH

Source/Database, host, date searched	Search strategy		Number of results
**CINAHL Plus with full text (EBSCOhost)**<br>September 26, 2020	#	Query	619
	S1	(MH "Urinary Tract Infections+")	
	S2	TI (urinary N3 infection* OR "urinary tract infection") OR AB (urinary N3 infection* OR "urinary tract infection")	
	S3	TI (uti OR CAUTI OR utis OR CAUTIs) OR AB (uti OR CAUTI OR utis OR CAUTIs)	
	S4	TI (cystitis OR pyelonephritis OR bacteriuria) OR AB (cystitis OR pyelonephritis OR bacteriuria)	
	S5	TI kidney N3 infection* OR AB kidney N3 infection*	
	S6	S1 OR S2 OR S3 OR S4 OR S5	
	S7	(MH "Nursing Homes+")	
	S8	(MH "Residential Facilities")	
	S9	(MH "Long Term Care")	
	S10	TI ((care OR residential OR nursing OR "aged care" OR geriatric) N3 (home* OR facilit*)) OR AB ((care OR residential OR nursing OR "aged care" OR geriatric) N3 (home* OR facilit*))	
	S11	TI (("long term" OR long-term OR longterm) N3 (care OR facilit* OR centre OR center OR institution*)) OR AB (("long term" OR long-	

	term OR longterm) N3 (care OR facilit* OR centre OR center OR institution*))<br>S12 TI ("residential care" OR "institutional care" OR "aged care facilities" OR "old* people* home*" OR "rest home*") OR AB ("residential care" OR "institutional care" OR "aged care facilities" OR "old* people* home*" OR "rest home*")<br>S13 TI ((care OR centre OR center) N3 day) OR AB ((care OR centre OR center) N3 day)<br>S14 S7 OR S8 OR S9 OR S10 OR S11 OR S12 OR S13<br>S15 S6 AND S14<br>S16 S6 AND S14<br>Limiters - Published Date: 20100101-20201231<br>S17 S6 AND S14<br>Limiters - Published Date: 20100101-20201231<br>Narrow by Language: - English	
**Ovid MEDLINE(R) and Epub Ahead of Print, In-Process & Other Non-Indexed Citations, Daily and Versions(R)** 1946 to September 25, 2020<br><br>Date searched: 26/09/2020	# Searches<br>1 exp Urinary Tract Infections/<br>2 ((urinary adj3 infection*) or "urinary tract infection*").tw.<br>3 (uti or CAUTI or utis or CAUTIs).tw.<br>4 (cystitis or pyelonephritis or bacteriuria).tw.<br>5 (kidney adj3 infection*).tw.<br>6 1 or 2 or 3 or 4 or 5<br>7 exp Nursing Homes/<br>8 exp Residential Facilities/<br>9 Long-Term Care/<br>10 ((care or residential or nursing or "aged care" or geriatric) adj3 (home* or facilit*)).tw.<br>11 (("long term" or long-term or longterm) adj3 (care or facilit* or centre or center or institution*)).tw.<br>12 ("residential care" or "institutional care" or "aged care facilities" or "old* people* home*" or "rest home*").tw.<br>13 ((care or centre or center) adj3 day).tw.<br>14 7 or 8 or 9 or 10 or 11 or 12 or 13<br>15 6 and 14<br>16 limit 15 to yr="2010 -Current"<br>17 limit 16 to english language	669

**Ovid Embase 1996 to 2020 Week 39**<br><br>Date searched: 26/09/2020	#	Searches	1870
	1	exp urinary tract infection/	
	2	((urinary adj3 infection*) or "urinary tract infection*").tw.	
	3	(uti or CAUTI or utis or CAUTIs).tw.	
	4	(cystitis or pyelonephritis or bacteriuria).tw.	
	5	(kidney adj3 infection*).tw.	
	6	1 or 2 or 3 or 4 or 5	
	7	nursing home/	
	8	residential home/	
	9	long term care/	
	10	home for the aged/	
	11	institutional care/	
	12	((care or residential or nursing or "aged care" or geriatric) adj3 (home* or facilit*)).tw.	
	13	(("long term" or long-term or longterm) adj3 (care or facilit* or centre or center or institution*)).tw.	
	14	("residential care" or "institutional care" or "aged care facilities" or "old* people* home*" or "rest home*").tw.	
	15	((care or centre or center) adj3 day).tw.	
	16	7 or 8 or 9 or 10 or 11 or 12 or 13 or 14 or 15	
	17	6 and 16	
	18	limit 17 to yr="2010 -Current"	
	19	limit 18 to english language	
**Cochrane Library**<br><br>Cochranelibrary.com<br><br>Date Run: 26/09/2020	ID	Search *Hits*	295
	#1	MeSH descriptor: [Urinary Tract Infections] explode all trees *2540*	
	#2	((urinary near/3 infection*) or "urinary tract infection*"):ti,ab,kw (Word variations have been searched) *8748*	
	#3	(uti or CAUTI or utis or CAUTIs):ti,ab,kw (Word variations have been searched) *2104*	
	#4	(cystitis or pyelonephritis or bacteriuria):ti,ab,kw (Word variations have been searched) *3382*	
	#5	(kidney near/3 infection*):ti,ab,kw (Word variations have been searched) *854*	
	#6	#1 or #2 or #3 or #4 or #5 *11834*	
	#7	MeSH descriptor: [Nursing Homes] explode all trees *1341*	
	#8	MeSH descriptor: [Residential Facilities] explode all trees *1745*	
	#9	MeSH descriptor: [Long-Term Care] this term only *1121*	

	#10 ((care or residential or nursing or "aged care" or geriatric) near/3 (home* or facilit*)):ti,ab,kw (Word variations have been searched) *15370*<br>#11 (("long term" or long-term or longterm) near/3 (care or facilit* or centre or center or institution*)):ti,ab,kw (Word variations have been searched) *7782*<br>#12 ("residential care" or "institutional care" or "aged care facilities" or "old* people* home*" or "rest home*"):ti,ab,kw (Word variations have been searched) *1120*<br>#13 ((care or centre or center) near/3 day):ti,ab,kw (Word variations have been searched) *3442*<br>#14 #7 or #8 or #9 or #10 or #11 or #12 or #13 *25475*<br>#15 #6 and #14 with Cochrane Library publication date Between Jan 2010 and Sep 2020 *295*	
**Bibliomap** https://eppi.ioe.ac.uk/ webdatabases/ SearchIntro.aspx<br><br>Date searched: 26/09/2020	#1 Characteristics of the study population: older people *2772*<br>#2 Intervention site(s): hospice OR residential care *89*<br>#4 Freetext: “urinary tract infection*” or uti or utis or cauti or cautis or cystitis or pyelonephritis or bacteriuria or “kidney infection*” *4*<br>#5 1 AND 2 AND 4 *0*<br>#6 2 AND 4 *0*<br>#7 1 AND 4 *0*	0
**Sociological Abstracts (via Proquest)**<br><br>Date searched: 01/10/2020	((ti((urinary NEAR/3 infection*) OR "urinary tract infection*") OR ab((urinary NEAR/3 infection*) OR "urinary tract infection*")) OR (ti(uti OR CAUTI OR utis OR CAUTIs) OR ab(uti OR CAUTI OR utis OR CAUTIs)) OR (ti(cystitis OR pyelonephritis OR bacteriuria) OR ab(cystitis OR pyelonephritis OR bacteriuria)) OR (ti(kidney NEAR/3 infection*) OR ab(kidney NEAR/3 infection*))) AND (MAINSUBJECT.EXACT("Nursing Homes") OR MAINSUBJECT.EXACT("Long Term Care") OR (ti((care OR residential OR nursing OR "aged care" OR geriatric) NEAR/3 (home* OR facilit*)) OR ab((care OR residential OR nursing OR "aged care" OR geriatric) NEAR/3 (home* OR facilit*))) OR (ti(("long term" OR long-term OR longterm) NEAR/3 (care OR facilit* OR centre OR center OR institution*)) OR ab(("long term" OR long-term OR longterm) NEAR/3 (care OR facilit* OR centre OR center OR institution*))) OR (ti("residential care" OR "institutional care" OR "aged care facilities" OR "old* people* home*" OR "rest home*") OR ab("residential care" OR "institutional care" OR "aged care facilities" OR "old* people* home*" OR "rest home*")) OR (ti((care OR centre OR center) NEAR/3 day) OR ab((care OR centre OR center) NEAR/3 day)))Limits applied<br><br>Databases:<br>Sociological Abstracts<br>Narrowed by year: 2014; 2016; 2017;<br>Language: English	

**Web of Science Core Collection**<br>*Indexes=SCI-EXPANDED, SSCI, A&HCI, CPCI-S, CPCI-SSH, BKCI-S, BKCI-SSH, ESCI, CCR-EXPANDED, IC Timespan=All years*<br><br>Date searched: 01/10/2020	#1 TOPIC: (urinary tract infection*)<br>#2 TI=(urinary NEAR/3 infection* OR “urinary tract infection”) OR AB=(urinary NEAR/3 infection* OR “urinary tract infection”)<br>#3 TI=(uti OR CAUTI OR utis OR CAUTIs) OR AB=(uti OR CAUTI OR utis OR CAUTIs)<br>#4 TI=(cystitis OR pyelonephritis OR bacteriuria) OR AB=(cystitis OR pyelonephritis OR bacteriuria)<br>#5 TI=(kidney NEAR/3 infection*) OR AB=(kidney NEAR/3 infection*)<br>#6 #1 or #2 or #3 or #4 or #5<br>#7 TS=("nursing home*" OR "residential facilit*" OR "skilled nursing facilit*" OR "long-term care" or "home for the aged")<br>#8 TI=((care or residential or nursing or "aged care" or geriatric) NEAR/3 (home* or facilit*)) OR AB=((care or residential or nursing or "aged care" or geriatric) NEAR/3 (home* or facilit*))<br>#9 TI=(("long term" or long-term or longterm) NEAR/3 (care or facilit* or centre or center or institution*)) OR AB=(("long term" or long-term or longterm) NEAR/3 (care or facilit* or centre or center or institution*))<br>#10 TI=("residential care" or "institutional care" or "aged care facilities" or "old* people* home*" or "rest home*") OR AB=("residential care" or "institutional care" or "aged care facilities" or "old* people* home*" or "rest home*")<br>#11 TI=((care or centre or center) NEAR/3 day) OR AB=((care or centre or center) NEAR/3 day)<br>#12 #7 or #8 or #9 or #10 or #11<br>#13 #6 and #12<br>#14 (#6 and #12) AND LANGUAGE: (English)<br>Indexes=SCI-EXPANDED, SSCI, A&HCI, CPCI-S, CPCI-SSH, BKCI-S, BKCI-SSH, ESCI, CCR-EXPANDED, **IC Timespan=2010-2020**	865
**NIHR Journals Library**<br>www.journalslibrary.nihr.ac.uk/<br><br>Date searched: 01/10/2020	("urinary tract infection*" or uti or cauti or cystitis or pyelonephritis or bacteriuria or "kidney infection*") AND ("nursing home*" or "care home*" or "residential home*" or "geriatric home*" or "rest home*" or "old* people" or "elder* care")<br>0 results<br>"urinary tract infection" AND "care home"<br>0 results<br>"urinary tract infection"<br>17 results	2

Two relevant studies:

- **StOP UTI**
- **Probiotic to Reduce Infections iN CarE home reSidentS (PRINCESS)**

  https://www.journalslibrary.nihr.ac.uk/programmes/eme/139510/#/

  Eleri Owen-Jones, Rachel Lowe, Mark Lown et al. **Probiotic to Reduce Infections iN CarE home reSidentS (PRINCESS) - Protocol paper** *BMJ Open* 2019
  Butler C, Lau, M, Gillespie G, Owen-Jones E, Lown M et al. **Effect of probiotic use on antibiotic administration among care home residents: a randomized clinical trial** *JAMA: Journal of the American Medical Association* 2020

Both of the PRINCESS study papers were retrieved in the other database searches.
"urinary tract infection" or "urinary tract infections"

4014 results

2011-2020: 1569 results

2021-2030: 290 results

"urinary tract infection" or "urinary tract infections" AND "older people"

0 results

“urinary tract infection” AND care home = 0 results

“urinary tract infection” AND old = 0 results

“urinary tract infection” AND elderly = 0 results

“urinary tract infection” AND geriatric = 0 results

"old people" or "older people" or elderly or "care home"

4067 results

Limited to HRCS health category: Renal and Urogenital

107 results – not finding UTIs

Exploratory AHRQ safety program search

**Source/Database, host, date searched**	**Search strategy**	**Number of results**
Collated publications of the AHRQ Safety Program for Long-Term Care		
**Scopus** Scopus.com<br>Date searched:<br>10/2020	• all fields: “AHRQ Safety Program for Long-Term Care” = 3 results, 2 new (1 already a key paper)<br>• combinations of title-abstract-key: “national collaborative” AND (“urinary tract infection*” or UTI or CAUTI) AND AHRQ AND affil: Michigan – no new relevant results.	2
**GoogleScholar**<br>Date searched:<br>10/2020	• "AHRQ Safety Program for Long-Term Care" 31 results, 4 new relevant. Found program name in references of articles and in the full text.	4
**Program final report**	https://www.ahrq.gov/sites/default/files/wysiwyg/professionals/quality-patient-safety/quality-resources/tools/cauti-ltc/modules/final-report.pdf<br><br>References	5

**Program website**<br><br>https://www.ahrq.gov/<br><br>hai/quality/tools/cauti-ltc/about-toolkit.html<br><br>Date searched: 10/2020	References on webpage = 6 – not program related publications<br><br>▪ 1. HHS National Action Plan to Prevent Health-care Associated Infections: Road Map to Elimination. Web-based guide. April 2013. U.S. Department of Health & Human Services. https://health.gov/hcq/prevent-hai-action-plan.asp. Accessed August 18, 2015.<br>▪ 2. HHS National Action Plan to Prevent Health-care Associated Infections: Road Map to Elimination. Web-based guide. April 2013. U.S. Department of Health & Human Services. https://health.gov/hcq/prevent-hai-action-plan.asp. Accessed August 18, 2015.<br>▪ 3. Richards CL. Infections in residents of long-term care facilities: an agenda for research. Report of an expert panel. J Am Geriatr Soc. 2002;50:570-6. PMID: 11943058.<br>▪ 4. Wagner, LM., Roup, B.J., Castle, NG. Impact of infection preventionists on Centers for Medicare and Medicaid quality measures in Maryland nursing homes. Am J Infect Control. 2014; Jan 42(1): 2-6. PMID: 24388467.<br>▪ 5. Smith PW, Bennett G, Bradley SF, et al. SHEA/APIC Guideline: Infection prevention and control in the long-term care facility. Infect Control Hosp Epidemiol. 2008; 29:785–814. PMID: 18786461.<br>▪ 6. Mody L, Bradley SF, Galecki A, et al. Conceptual model for reducing infections and antimicrobial resistance in skilled nursing facilities: focus on residents with indwelling devices. Clin Infect Dis. 2011;52:654-61. PMID: 21292670.	6

Citation searching of 8 key papers

Scopus Scopus.com
And
Google Scholar

Date searched:
10/2020

391

Paper	Forward citations Scopus (Scholar) [cited by]	Backward citations Scopus (of which papers published within the last ten years) [references]	Notes
Adkins J. Participating in a national project, Pennsylvania nursing homes reduce CAUTI. Patient Saf Advis 2016; Dec; 13(4): 149-153.	0 (0)	0 (0)	Not indexed in Scopus. Carried out a Reference field search for both title and the URL.
Bradley S. Evaluating the effect of infection control practices on reduction of CAUTIs in Pennsylvania	0 (0)	0 (0)	Not indexed in Scopus.<br><br>Carried out a Reference field search for

Long-term care facilities. Patient Saf Advis 2016 Dec; 13(4): 154-159.			both title and the URL.
Hutton DW et al. Economic evaluation of a catheter-associated urinary tract program in nursing homes. J Am Ger Soc 2018; 66(4): 742-747.	5 (13)	29 (20)	
Krein SL, Harrod M, Collier S et al. A national collaborative approach to reduce catheter-associated urinary tract infections in nursing homes: A qualitative assessment. Am J Infect Control 2017; 45(12): 1342-1348.	4 (9)	26 (22)	
Krein SL, Greene M, King B et al. Assessing a National Collaborative Program To Prevent Catheter-Associated Urinary Tract Infection in a Veterans Health Administration Nursing Home Cohort. Infect Control Hosp Epidemiol 2018; 39(7): 820-825.	4 (4)	24 (20)	
Meddings J, Saint S, Krein S et al.	18 (23)	115 (50)	

	Systematic review of interventions to reduce urinary tract infection in nursing home residents. J Hosp Med 2017; 12(5): 365-8.			
	Mody L, Meddings J, Edson BS et al. Enhancing resident safety by preventing healthcare-associated infection: A national initiative to reduce catheter-associated urinary tract infections in nursing homes. CID 2015; 61(1):86-94.	25 (48)	53 (21)	
	Mody L, Greene TM, Meddings J et al. A national implementation project to prevent catheter-associated urinary tract infection in nursing home residents. JAMA Intern Med 2017; 177(8): 1154-1162.	44 (57)	41 (28)	
**Publish or Perish**	Adkins 2016 0<br>Bradley 2016 0<br>Hutton 2018 10<br>Krein 2017 7<br>Krein 2018 4<br>Meddings 2017 22<br>Mody 2015 37			134

	Mody 2017 54	

Key authors' publications

Author institution webpage and Scopus	Krein = total 153<br>Meddings = total 77<br>Mody = total 211<br>Saint = total 240	681
