# Supplementary material for: Preventing urinary tract infection in older people living in care homes: the ‘StOP UTI’ realist synthesis
Source: BMJ Qual Saf. 2024 Aug 8;34(3):e016967. doi: 10.1136/bmjqs-2023-016967 (PMC11874410; doi:10.1136/bmjqs-2023-016967)
Supplement: online supplemental file 2 [file bmjqs-34-3-s002.pdf]

Supplementary File 2: List of 'If…then' statements

Hydration

- If staff are encouraged to focus on resident hydration then they will be more confident that they can support them to drink. (Booth, Lean, Wilson)
- If staff are more aware of the importance of hydration, need to increase opportunities and choice of drinks, then residents will be supported to drink more fluids. (Booth, Lean, Wilson)
- If more drinking opportunities are incorporated into daily activities, then staff can devote more time to supporting residents to drink and the frequency of UTI among residents decreases. (Booth, Lean, Wilson)
- If fluid intake targets are set, then the residents will be supported to drink more fluids. (Booth, Lean, Wilson)
- If drinking rounds are monitored by drinks champion, then they will be delivered. (Booth, Lean, Wilson)

Preventing recurrent urinary tract infection (UTI)

- If care home residents are identified (using assessments, through review of care and medical notes, from recurrent UTI, from other conditions/characteristics known to increase risk of UTI, from catheter use) as potentially benefiting from the use of non-antibacterial prophylaxis (cranberry capsules, herbal teas, oestrogen treatment) and use of the prophylaxis is seen as a routine and important part of their care (correct dosage, timing of administration, recording/reporting of administration) then it is likely there will be a reduction in UTI.

Catheter-associated urinary tract infection (CAUTI) prevention

- If educational material is available in a range of formats and delivered by knowledgeable practitioners in a way that fits workforce abilities, then practice is improved.
- If an improvement programme addresses not only knowledge barriers, but also a broad range of behavioural constructs to support change (e.g. beliefs, emotions, reinforcement, environmental context and resources) and aligns with external programmes and services then it is more likely to be effective.
- If care home staff are trained to implement evidence-based CAUTI interventions, incontinence planning and hydration activity they are empowered to challenge requests to catheterise and order urine cultures. (Mody 2017)
- If care home staff (nurses and healthcare support workers) are trained to implement evidence-based CAUTI interventions, incontinence planning and hydration activity, then rates of CAUTI will be minimised (practice will be enhanced). (Mody 2017)
- If care home staff have access to skilled facilitation (coaching) implementation of evidence-based CAUTI interventions, incontinence planning and hydration activity then improvement is more likely to succeed.
- If a collaborative approach is used with feedback to care home staff, then they are motivated to adhere to best practice.

Understanding what constitutes a UTI

- If care home staff understand the signs and symptoms of UTI in their residents and can communicate key clinical observations within their team and to primary care staff (common language and common understanding of what needs to be communicated), then the accuracy of UTI diagnosis will be improved. (Arnold 2020)
- If there is a common understanding of signs and symptoms of UTI (through education and use of language that is appropriate for the level of clinical knowledge) then it is likely appropriate information will be communicated accurately across the care home and health care team. (Arnold 2020)
- If an improvement programme addresses not only knowledge barriers, but also a broad range of behavioural constructs to support change (e.g. beliefs, emotions, reinforcement, environmental context and resources) and aligns with external programmes and services then it is more likely to be effective. (Chambers 2018; Pasay 2019)

Recognising UTI and communicating concerns

- If the responsibilities of the care home staff in the recognition and diagnosis process of UTI for their residents is understood and they feel confident in raising and communicating concerns with colleagues then potential UTIs are likely to be investigated and treated. (Arnold 2020)
- If care home staff (nurses and healthcare support workers) are actively involved in assessing and recognising early signs and symptoms suggestive of UTI and physicians have confidence in the information they provide, then opportunities to instigate preventative measures (e.g. increased oral hydration, support with hygiene, support with toileting) and avoid unnecessary antibiotic treatment are more likely to be applied.
- If there is a common understanding of signs and symptoms of UTI (through education and use of language that is appropriate for the level of clinical knowledge) then it is likely appropriate information will be communicated accurately across the care home and health care team.
- If an algorithm is a good fit with actual situations in practice and supports communication by care staff with clear roles and responsibilities, then it may promote use of preventative measures (e.g. encouraging fluid intake) before contacting the doctor for a suspected infection. (Potter 2019; Hughes 2020; Pasay 2019)
- If interventions for improving UTI recognition and diagnosis are designed with those involved (care home staff and primary care staff) then interventions are more likely to be used and be useful because they identify and overcome the challenges for implementation.
- If assessment/decision tools allow for systematic gathering and structuring of relevant information (signs and symptoms that may or may not relate to UTI) that can be used to assess and communicate the likelihood someone has a UTI (through evidence-based algorithms) and are considered easy to use and useful to those using them, then they can improve recognition/diagnosis of UTI. (Arnold 2020)
- Digital support tools could help care assistants make good decisions or re-enforce good decision making. Tools need to provide rapid responsive information and be appropriate for existing technology (Jones 2017)

Active monitoring

- If residents and family members are engaged on the topic of AMR, better informed about alternative preventative strategies to minimise risk of UTI and involved in decisions, then they may be more supportive of efforts to reduce use of antibiotics. (Potter 2019; Chambers 2018; Pasay 2019)
- If care home staff (nurses and healthcare support workers) are actively involved in assessing and recognising early signs and symptoms suggestive of UTI and physicians have confidence in the information they provide, then opportunities to instigate preventative measures (e.g. increased oral hydration, support with hygiene, support with toileting) and avoid unnecessary antibiotic treatment are more likely to be applied. (Chaaban 2019; Tingstrom 2010)

Involving the resident and family

- If interventions for improving UTI recognition and diagnosis are designed with those involved (care home staff and primary care staff) then interventions are more likely to be used and be useful because they identify and overcome the challenges for implementation. (Arnold 2020)
- If an intervention is multidisciplinary (addressing the nurse–physician–family triad), incorporates audit and feedback and is integrated into existing quality, safety and infection prevention and control programmes, then this may be more influential in reducing unnecessary antibiotic prescribing in care home settings.
- If residents and families are better informed about alternative preventative strategies to minimise risk of UTI, they may be more supportive of efforts to reduce use of antibiotics.
- If family members are engaged on the topic of antimicrobial resistance and involved in decisions about ill residents, then this may build confidence and trust, promoting partnership working.
- If care home staff have access to skilled facilitation (coaching) implementation of evidence-based CAUTI interventions, incontinence planning and hydration activity then improvement is more likely to succeed. (Mody 2017)
- If educational material is available in a range of formats and delivered by knowledgeable practitioners in a way that fits workforce abilities, then practice is improved. (Mody 2017)
- If a collaborative approach is used with feedback to care home staff, then they are motivated to adhere to best practice. (Mody 2017)
