# Supplementary material for: Preventing urinary tract infection in older people living in care homes: the ‘StOP UTI’ realist synthesis
Source: BMJ Qual Saf. 2024 Aug 8;34(3):e016967. doi: 10.1136/bmjqs-2023-016967 (PMC11874410; doi:10.1136/bmjqs-2023-016967)
Supplement: online supplemental file 3 [file bmjqs-34-3-s003.pdf]

Supplementary File 3: Summary of supplementary searches in Stage 2

Databases searched: OVID Medline, CINAHL Plus, OVID Embase, Cochrane Library, Web of Science Core Collection, Sociological Abstracts via Proquest, Bibliomap and NIHR Journals Library, OpenGrey, NHS Evidence, Social Care Online, Websites of relevant organisations.

Soft signs and patient deterioration

Highly focused searches to identify relevant results only. Ignored results to do with recognising soft signs in hospitals, and results to do with the NEWS tool for measurable signs, i.e. not soft signs.

Source/Database, host, date searched	Search strategy	Number of results
**RESTORE2 webpages** on AHSN website and **Google searches**<br>Date searched: June 2021	Iterative searching, by following links from the RESTORE2 webpages, and Google searches including the following keywords: patient and deteriorat*, care home, evaluation. Searching for specific tools as they were identified (e.g. Significant Care, Significant 7, Stop and Watch, Is my resident unwell?), "soft sign*"	31
**CINAHL (EBSCOhost)**<br>Date searched: 05/07/2021	S1 TI "soft sign*" OR AB "soft sign*"<br><br>S2 TI (deteriorat* or "care home*" or "nursing home*" or infection* or older or elderly or resident*) OR AB (deteriorat* or "care home*" or "nursing home*" or infection* or older or elderly or resident*)<br><br>S3 S1 AND S2<br><br>10 results, 0 relevant.	0
**Ovid MEDLINE(R) and Epub Ahead of Print, In-Process, In-Data-Review & Other Non-Indexed Citations, Daily and Versions(R)** 1946 to July 02, 2021<br>Date searched: 05/07/2021	1 "soft sign*".tw.<br>2 (deteriorat* or "care home*" or "nursing home*" or infection* or older or elderly or resident*).tw.<br>3 1 and 2<br>38 results, 0 relevant to UTI or infections in care homes	0
**Open Grey**<br>Date searched: 05/07/2021	"soft sign" = 0 results<br>"soft signs" = 1 result, 0 relevant (dyslexia)	0

Non-antimicrobial therapeutic interventions for recurrent UTI, including oestrogen therapy

Highly focused searches to identify relevant results only.

Source/Database, host, date searched	Search strategy	Number of results
Reference checking of the following key review publications.<br><br>Date searched: September 2021	1. NICE Guideline: Urinary tract infection (recurrent): antimicrobial prescribing. NG112 (31 October 2018). (14 references). Available at https://www.nice.org.uk/guidance/ng112<br>2. Perrotta C, Aznar MM, Albert X and Ng CW. Oestrogens for preventing recurrent UTI in postmenopausal women. Cochrane Database of Systematic Reviews 2008, Issue 2.Art. No.: CD005131. (9 references)	13

	3. Sihra N, Goodman A, Zakri R et al. Nonantibiotic prevention and management of recurrent urinary tract infection. Nature Reviews Urology 2018; Oct 25:1. (248 references)	
Added after consultation with experts	Bonkat et al., 2022 - EUA guidelines<br>Harding – Hippurate trial results (2022)	2

Hydration to prevent UTI

Date limit of five years set to retrieve only the most recent publications as one of the research team had already undertaken a previous systematic search.

**Source/Database, host, date searched**	**Search strategy**		**Number of results**
**Ovid MEDLINE(R) and Epub Ahead of Print, In-Process, In-Data-Review & Other Non-Indexed Citations, Daily and Versions(R)** 1946 to November 29, 2021<br><br>Date searched: 30/11/2021	#	Searches	56
	1	exp Urinary Tract Infections/pc	
	2	("urinary tract infection*" adj15 (prevent* or reduc* or increas*)).tw.	
	3	((bacteriuria* or pyuria*) adj15 (prevent* or reduc* or increas*)).tw.	
	4	1 or 2 or 3	
	5	Dehydration/	
	6	dehydrat*.tw.	
	7	hydrat*.tw.	
	8	Drinking/	
	9	drinking.tw.	
	10	((water or fluid*) adj3 (intake or consumption)).tw.	
	11	water-electrolyte balance/	
	12	fluid therapy/	
	13	5 or 6 or 7 or 8 or 9 or 10 or 11 or 12	
	14	4 and 13	
	15	limit 14 to yr="2016 -Current"	
	16	limit 15 to english language	
**CINAHL (EBSCOhost)** November 30, 2021<br><br>Date searched: 30/11/2021	#	Query	49
	S1	(MH "Urinary Tract Infections/PC")	
	S2	TI ("urinary tract infection*" N15 (prevent* or reduc* or increas*)) OR AB ("urinary tract infection*" N15 (prevent* or reduc* or increas*))	
	S3	TI ((bacteriuria* or pyuria*) N15 (prevent* or reduc* or increas*)) OR AB ((bacteriuria* or pyuria*) N15 (prevent* or reduc* or increas*))	
	S4	S1 OR S2 OR S3	
	S5	MH "Hydration Status"	
	S6	MH "Dehydration"	
	S7	MH "Fluid Therapy"	
	S8	TI hydrotherapy OR AB hydrat*	

	S9 TI (dehydration or fluid deficit or lack of fluid or fluid deficiency) OR AB dehydrat*<br>S10 TI drinking OR AB drinking<br>S11 TI ((water or fluid*) N3 (intake or consumption)) OR AB ((water or fluid*) N3 (intake or consumption))<br>S12 S5 OR S6 OR S7 OR S8 OR S9 OR S10 OR S11<br>S13 S4 AND S12<br>S14 S4 AND S12 Limiters - Published Date: 20160101-20211231<br>S15 S4 AND S12 Narrow by Language: - english	
**SCIE Social Care Online**	Subject term “hydration” include this term only AND all fields “urinary tract infection” = 0 results<br><br>Subject term “hydration” include this term only AND all fields “infection” = 2 results, 2015-current = 0 results.<br><br>Subject term: “older people” include narrower terms AND subject term: “hydration” = 29 results, 2016-current = 7 results	7
**Citation searching of key papers using Scopus**		
**Scopus**<br><br>Date searched: 30/11/2021	Citation search of Wilson 2019<br>Reference search of Wilson 2019 (only include 2016 onwards)<br>Citation search of Scott 2020<br>Reference search of Scott 2020 (only include 2016 onwards)	8<br>4<br>5<br>8
**Other grey literature**		
**SCIE website**<br>Date searched: 30/11/2021	https://www.scie.org.uk/search?sq=hydration+AND+urinary+tract+infection<br>4 results, 3 relevant (2 are the same resource)	2
**NHS Improvement website:**<br>England.nhs.uk<br>Date searched: 30/11/2021	0 results for both urinary tract infection AND hydration	0
**AHSN Network:**<br>ahsnnetwork.com<br>Date searched: 30/11/2021	1 result for hydration or hydrate (Good Hydration! project)	1
**Open Grey**<br>Date searched: 02/12/2021	Hydrat* AND “urinary tract infection*” = 0 results; hydrat* AND infection* = 0 results; “urinary tract infection*” = 8 results, none on prevention or older people = 0.	0
**Google searches**<br>Date searched: 01-02/12/2021	(hydrate OR hydration) AND "urinary tract infection" AND site:.nhs.uk<br>About 3,860 results, first 10 pages browsed = 11 results<br><br>(hydrate OR hydration) AND "urinary tract infection" AND site:.gov.uk<br>About 537 results, first 5 pages browsed = 4 results<br><br>(hydrate OR hydration) AND "urinary tract infection" AND site:.org.uk<br>About 1,440 results, first 5 pages browsed = 4 results<br><br>(hydrate OR hydration) AND "urinary tract infection" AND site:.ac.uk	

	About 2,990 results, first 5 pages browsed = 1 result	
**RCN** Rcn.org.uk<br>Date searched: 02/12/2021	search box: urinary tract infection<br>3 results, none relevant<br>Search box: hydration<br>22 results, none relevant	0
**AGE UK** Ageuk.org.uk<br>Date searched: 02/12/2021	search box: urinary tract infections<br>0 results linking hydration and UTIs	0
**CQC** cqc.org.uk<br>Date searched: 02/12/2021	Search themed publications (n=47), 0 relevant results	0

Family involvement

A date limit of 10 years was applied in line with the main search.

**Source/Database, host, date searched**	**Search strategy**		**Number of results**
**Ovid MEDLINE(R) and Epub Ahead of Print, In-Process, In-Data-Review & Other Non-Indexed Citations, Daily and Versions(R)** 1946 to December 02, 2021<br><br>Date searched: 03/12/2021	#	Searches	303
	1	Family/	
	2	Caregivers/	
	3	(family or families or spouse* or "care partner*" or relatives or relation or relations).tw.	
	4	1 or 2 or 3	
	5	Decision Making, Shared/	
	6	Decision Making/	
	7	decision?making.tw.	
	8	((care or diagnosis or treatment) adj3 (decision* or participat* or involve* or influenc* or engage*)).tw.	
	9	((notic* or detect*) adj3 (signs or symptoms or change* or deterioration)).tw.	
	10	5 or 6 or 7 or 8 or 9	
	11	4 and 10	
	12	Professional-Family Relations/	
	13	((family or families*) adj3 (involve* or influenc* or participat* or engage*)).tw.	
	14	12 or 13	
	15	11 or 14	
	16	Homes for the Aged/	
	17	"care home*".tw.	
	18	("care setting*" adj12 ("old* people" or elderly or dementia)).tw.	
	19	("nursing home*" adj12 ("old* people" or elderly or dementia)).tw.	
	20	("social care" and ("old* people" or elderly or dementia)).tw.	
	21	16 or 17 or 18 or 19 or 20	

	22	exp Urinary Tract Infections/	
	23	"urinary tract infection*".tw.	
	24	(bacteriuria* or pyuria*).tw.	
	25	22 or 23 or 24	
	26	15 and 21	
	27	15 and 25	
	28	26 or 27	
	29	Terminal Care/	
	30	("terminal care" or "end of life" or end-of-life).tw.	
	31	29 or 30	
	32	28 not 31	
	33	(paediatric or pediatric or child* or teenage* or adolescent* or baby or babies or newborn* or infant*).tw.	
	34	32 not 33	
	35	limit 34 to yr="2012 -Current"	
	36	limit 35 to english language	
**CINAHL (EBSCOhost)** December 03, 2021	#	Query	63
	S1	(MH "Nuclear Family+") OR (MH "Extended Family") OR (MH "Family")	
	S2	(MH "Caregivers")	
	S3	TI (family or families or spouse* or "care partner*" or relatives or relation or relations) OR AB (family or families or spouse* or "care partner*" or relatives or relation or relations)	
	S4	S1 OR S2 OR S3	
	S5	(MH "Decision Making, Shared")	
	S6	(MH "Decision Making")	
	S7	TI decision*making OR AB decision*making	
	S8	TI ((care or diagnosis or treatment) N3 (decision* or participat* or involve* or influenc* or engage*)) OR AB ((care or diagnosis or treatment) N3 (decision* or participat* or involve* or influenc* or engage*))	
	S9	TI ((notic* or detect*) N3 (signs or symptoms or change* or deterioration)) OR AB ((notic* or detect*) N3 (signs or symptoms or change* or deterioration))	
	S10	S5 OR S6 OR S7 OR S8 OR S9	
	S11	S4 AND S10	
	S12	(MH "Professional-Family Relations")	
	S13	(MH "Decision Making, Family")	

	S14 (MH "Patient-Family Conferences")<br>S15 TI ((family or families*) N3 (involve* or influenc* or participat* or engage*)) OR AB ((family or families*) N3 (involve* or influenc* or participat* or engage*))<br>S16 S12 OR S13 OR S14 OR S15<br>S17 S11 OR S16<br>S18 TI ("nursing home*" N12 ("old* people" or elderly)) OR ("nursing home*" N12 ("old* people" or elderly))<br>S19 TI ("care home*" N12 ("old* people" or elderly)) OR ("care home*" N12 ("old* people" or elderly))<br>S20 S18 OR S19<br>S21 (MH "Urinary Tract Infections+")<br>S22 TI (“urinary tract infection*”) OR AB (“urinary tract infection*”)<br>S23 TI (bacteriuria* or pyuria*) OR AB (bacteriuria* or pyuria*)<br>S24 S21 OR S22 OR S23<br>S25 S17 AND S20<br>S26 S17 AND S24<br>S27 S25 OR S26<br>S28 (MH "Terminal Care+")<br>S29 TI ("terminal care" or "end of life" or end-of-life) OR AB ("terminal care" or "end of life" or end-of-life)<br>S30 S28 OR S29<br>S31 S27 NOT S30<br>S32 TI (paediatric or pediatric or child* or teenage* or adolescent* or baby or babies or newborn* or infant*) OR AB (paediatric or pediatric or child* or teenage* or adolescent* or baby or babies or newborn* or infant*)<br>S33 S31 NOT S32<br>S34 S31 NOT S32 Limiters - Published Date: 20120101-20211231<br>S35 S31 NOT S32 Narrow by Language: - english	
**SCIE Social Care Online**<br>Date searched: 03/12/2021	Subject term “families” AND subject term “care homes” NOT title “visit*”: 172 records<br>limited to 2012 onwards: **56** records<br><br>Subject term “families” AND subject term “infectious diseases” NOT title “visit*” – 10 results, none relevant<br>All fields: “urinary tract infection*” AND subject term “families” = 0 results.	56
**NICE Evidence**<br>Date searched: 03/12/2021	Various iterations of the following search terms: family, families, care homes, family participation, family engagement, family involvement, family influence	0

	1 potentially relevant result – also identified in Social Care Online (Honoured guests. 2017)<br>Other results were to do with the impact of visiting restrictions during the recent pandemic and not to do with involvement in care of the resident.	
**Citation searching of key papers using Scopus**		
**Scopus**<br>Date searched: 03/12/2021	Mangal 2021 citations<br>Mangal S, Pho A, Arcia A, Carter E. Patient and family engagement in catheter-associated urinary tract infection (CAUTI) prevention: a systematic review. Jt Comm J Qual Patient Saf 2021; 47(9): 591-603.	1
	Mangal 2021 references (within 10-year date limit)	31
	Powell 2018 citations<br>Powell C, Blighe A, Froggatt K et al. Family involvement in timely detection of changes in health of nursing home residents: A qualitative exploratory study. J Clin Nurse 2018; 27(1-2): 317-327.	15
	Powell 2018 references (within 10-year date limit)	21
**Other grey literature**		
**SCIE (Social Care Institute for Excellence)**<br>Date searched: 03/12/2021	Scie.org.uk > subject topic 'Care homes' – 69 resources browsed, 1 relevant result	1
**Open Grey**<br>Date searched: 03/12/2021	Various combinations of keywords family, families, engagement, involvement, participation, care home* or urinary tract infection* or infection* = 0 results	0
**NHS Improvement**<br>England.nhs.uk<br>Date searched: 03/12/2021	Various combinations of keywords family, families, engagement, involvement, participation, care home* or urinary tract infection* or infection* = 0 results	0
**Age UK**<br>Date searched: 03/12/2021	Hand searched Reports and briefings, Consultation responses, and Evaluation reports = 0 results for family involvement in decision making for UTIs, infection or in care homes	0
**CQC** cqc.org.uk<br>Date searched: 03/12/2021	Hand search of publications section – 1 results	1
**Google searches**<br>Date searched: 03/12/2021	(family engagement) AND ("care home*") AND "care decisions" AND (site:.org.uk OR site:.nhs.uk OR site:.gov.uk)<br>About 2,360 results, first 5 pages browsed – 0 relevant results<br><br>(family involvement) AND ("care home*") AND "care decisions" AND (site:.org.uk OR site:.nhs.uk OR site:.gov.uk)<br>About 2, 250 results, first 5 pages browsed – 2 potentially relevant results.<br><br>(family OR families) AND ("care home*") AND (decision* OR involvement) AND (site:.org.uk OR site:.nhs.uk OR site:.gov.uk)<br>About 219,000 results, first 5 pages browsed – 0 relevant results	2

Continence and UTI

**Source/Database, host, date searched**	**Search strategy**		**Number of results**
**Ovid MEDLINE(R) and Epub Ahead of Print, In-Process, In-Data-Review &**	#	Searches	170
	1	exp *Urinary Incontinence/co, nu, pc, th [Complications, Nursing, Prevention & Control, Therapy]	

**Other Non-Indexed Citations, Daily and Versions(R)** 1946 to December 08, 2021<br><br>Date searched: 09/12/2021	2	((continence or incontinence or bladder or urine or urinary) adj3 (care or management or control)).tw.	
	3	Incontinence Pads/	
	4	"incontinence pad*".tw.	
	5	"urinary pad*".tw.	
	6	"continence aid*".tw.	
	7	"incontinence aid*".tw.	
	8	((avoid* adj3 catheter*) and (urine or urinary)).tw.	
	9	1 or 2 or 3 or 4 or 5 or 6 or 7 or 8	
	10	exp Urinary Tract Infections/	
	11	"urinary tract infection*".tw.	
	12	(bacteriuria* or pyuria*).tw.	
	13	Urinary Catheters/ae [Adverse Effects]	
	14	Urinary Catheterization/ae [Adverse Effects]	
	15	10 or 11 or 12 or 13 or 14	
	16	Nurse Specialists/	
	17	Nurse Clinicians/	
	18	Nurse Practitioners/	
	19	16 or 17 or 18	
	20	Urinary Incontinence/	
	21	19 and 20	
	22	(continence adj1 (advisor or advisors or nurse or nurses or specialist or specialists)).tw.	
	23	21 or 22	
	24	Homes for the Aged/	
	25	Nursing Homes/	
	26	Residential Facilities/	
	27	25 or 26	
	28	exp Aged/	
	29	27 and 28	
	30	(("care home*" or "care setting*" or "nursing home*" or "social care") adj12 ("old* people" or elderly)).tw.	
	31	24 or 29 or 30	
	32	exp *Aged/	
	33	("old* people" or elder*).tw.	
	34	32 or 33	
	35	9 and 15 and 31	
	36	9 and 15 and 34	
	37	15 and 23	
	38	23 and 31	
	39	23 and 33	
	40	35 or 36 or 37 or 38 or 39	
	41	limit 40 to english language	
**CINAHL Plus with Full text (EBSCOhost)**<br><br>Date searched: 09/12/2021	#	Query	142
	S1	(MH "Urinary Incontinence/CO/NU/PC/TH")	
	S2	(MH "Wound, Ostomy and Continence Nursing")	
	S3	TI ((continence or incontinence or bladder or urine or urinary) N3 (care or management or control)) OR AB ((continence or incontinence or bladder or urine or urinary) N3 (care or management or control))	
	S4	(MH "Incontinence Aids")	

	S5 TI ("incontinence pad*" or "urinary pad*") OR AB ("incontinence pad*" or "urinary pad*")<br>S6 TI ("continence aid*" or "incontinence aid*") OR AB ("continence aid*" or "incontinence aid*")<br>S7 TI ((avoid* N3 catheter*) and (urine or urinary)) OR AB ((avoid* N3 catheter*) and (urine or urinary))<br>S8 S1 OR S2 OR S3 OR S4 OR S5 OR S6 OR S7<br>S9 (MH "Urinary Tract Infections+")<br>S10 TI "urinary tract infection*" OR AB "urinary tract infection*"<br>S11 TI (bacteriuria* or pyuria*) OR AB (bacteriuria* or pyuria*)<br>S12 (MH "Catheters, Urinary+/AE")<br>S13 (MH "Urinary Catheterization+/AE")<br>S14 S9 OR S10 OR S11 OR S12 OR S13<br>S15 (MH "Continence Advisors")<br>S16 TI (continence N1 (advisor or advisors or nurse or nurses or specialist or specialists)) OR AB (continence N1 (advisor or advisors or nurse or nurses or specialist or specialists))<br>S17 S15 OR S16<br>S18 TI ("care home*" N12 ("old* people" or elderly)) OR AB ("care home*" N12 ("old* people" or elderly))<br>S19 TI ("nursing home*" N12 ("old* people" or elderly)) OR AB ("nursing home*" N12 ("old* people" or elderly))<br>S20 S18 OR S19<br>S21 (MM "Aged+")<br>S22 TI ("old* people" or elder*) OR AB ("old* people" or elder*)<br>S23 S21 OR S22<br>S24 S8 AND S14 AND S20<br>S25 S8 AND S14 AND S23<br>S26 S14 AND S17<br>S27 S17 AND S20<br>S28 S17 AND S23<br>S29 S24 OR S25 OR S26 OR S27 OR S28<br>S30 S24 OR S25 OR S26 OR S27 OR S28 Narrow by Language: - english	
**SCIE Social Care Online**<br><br>Date searched: 09/12/2021	Subject term: “incontinence” AND All fields: “urinary tract infections*” AND Subject term: “care homes” = 0 results	2

	All fields: “continence” AND All fields: “urinary tract infections*” AND Subject term: “care homes” = **1 result** (public reporting of quality indicators)<br>Subject term: “incontinence” AND All fields: “urinary tract infections*” AND Subject term: “older people” = 0 results<br>All fields: “continence” AND All fields: “urinary tract infections*” AND Subject term: “older people” = 0 results<br>All fields: “continence advisor*” OR All fields: “continence specialist*” = 0 results<br>Subject term: “incontinence” AND All fields: “nurse specialist*” = 0 results<br>Subject term: “incontinence” AND All fields: “nurse practitioner*” = **1 result** (continence care for people with dementia at home)	
**Other grey literature**		
**SCIE website**<br>www.scie.org.uk<br>Date searched: 08/12/2021	45 Results for continence, 2 relevant<br>89 results for incontinence, 0 relevant<br>4 results for continence advisor, 1 relevant	3
**NHS Improvement website:**<br>England.nhs.uk<br>Date searched: 08/12/2021	33 publication results for continence, 0 relevant<br>0 publication results for incontinence<br>0 results for continence advisor	0
**AHSN Network:**<br>ahsnnetwork.com<br>Date searched: 08/12/2021	0 results continence, incontinence, advisor	0
**Open Grey (DANS Easy Archive)**<br>Date searched: 08/12/2021	3 results for continence, 0 relevant	0
**RCN**<br>Rcn.org.uk<br>Date searched: 08/12/2021	Search box: continence<br>15 results, 1 relevant<br>Search box: Incontinence<br>14 results, 0 relevant<br>Search box: Continence advisor – 0 results<br>Search box: Continence specialist – 0 results	1
**AGE UK** Ageuk.org.uk<br>Date searched: 08/12/2021	Search box: continence<br>37 results, 1 relevant<br>Search box: incontinence<br>58 results, 0 relevant	1
**CQC**<br>cqc.org.uk<br>Date searched: 08/12/21	22 results for continence, 0 relevant<br>14 results for incontinence, 0 relevant	0
**Google searches**<br>Date searched: 09/12/2021	“continence care” AND “urinary tract infection*” AND "care home*" AND (site:.org.uk OR site:.nhs.uk OR site:.gov.uk)<br>About 337 results, first 5 pages screened, 4 results	8

(“continence advisor" or "continence specialist") AND ("care home*")
About 214 results, first 5 pages screened, 4 results
