# Supplementary material for: Preventing urinary tract infection in older people living in care homes: the ‘StOP UTI’ realist synthesis
Source: BMJ Qual Saf. 2024 Aug 8;34(3):e016967. doi: 10.1136/bmjqs-2023-016967 (PMC11874410; doi:10.1136/bmjqs-2023-016967)
Supplement: online supplemental file 4 [file bmjqs-34-3-s004.pdf]

Supplementary File 4: Figure 5 Relevant and good enough flowchart

Study ID / Author / Date
#

1. Does the evidence link to one or more of the study's programme theory areas?

Does it match?

('Fidelity')

Comments:

Evidence on theory area 1: nature of the intervention

yes / no

Evidence on theory area 2: facilitating and embedding good practice

yes / no

Evidence on theory area 3: care home context and culture

yes / no

Evidence on theory area 4: co-design and multi-agency working

yes / no

In your judgement:

2. Does the evidence provide valuable information? ("nuggets")

3. Can the evidence be relied upon? ("trustworthiness" – consider issues of sample size, data collection, data analysis and claims made)

4. Is the evidence what is needed? Does it contribute to the study? ("relevance")

Flowchart adapted from:
Williams L, Rycroft-Malone J, Burton CR *et al*. Improving skills and care standards in the support workforce for older people: a realist synthesis of workforce development interventions. BMJ Open 2016; 6:e011964.
