# Supplementary material for: Preventing urinary tract infection in older people living in care homes: the ‘StOP UTI’ realist synthesis
Source: BMJ Qual Saf. 2024 Aug 8;34(3):e016967. doi: 10.1136/bmjqs-2023-016967 (PMC11874410; doi:10.1136/bmjqs-2023-016967)
Supplement: online supplemental file 5 [file bmjqs-34-3-s005.pdf]

Supplementary File 5: Table 2 Data extraction form – completed example

**Study ID and full reference**	
#	3964
Document type	Journal article
What is the study's aim/purpose?	Paper describes the development of a tailored, complex intervention for a cluster-randomised trial that targets the knowledge of UTI and communication skills in nursing home staff to reduce antibiotic prescriptions.
What is the intervention (if applicable)?	Pre-intervention - development of a reflection tool (decision aid) and a communication tool (ISBAR), collectively referred to as a "dialogue tool", together with a case-based education session to support nursing home staff to convey relevant information to the GP on their observations of residents whom they consider may have a UTI to reduce misdiagnosis and unnecessary prescribing of antibiotics.
Design/method and its appropriateness for the aims/ purpose of the study	Mixed methods including participatory observations in nursing homes, interviews with stakeholders and survey of GPs to inform the design of a tailored complex intervention and identify and address barriers to implementation.<br>Design/method appropriate for the aims/purpose of the study.
Sample type and size	Observations in nursing home; Focus group with 3 healthcare helpers and 2 healthcare assistants; 'double' interview with 1 GP and 1 medical secretary; 'double' interview with 2 senior citizens; individual interviews with 2 registered nurses; 2 single interviews then 4 short telephone interviews with a head nurse and nurse involved in the pilot study. Purposive sampling.
Population and setting	Healthcare helpers and assistants working in nursing homes in Denmark, other key stakeholders including registered nurses, a GP, a medical secretary and two senior citizens.
Data collection method	Participatory observations in nursing homes; interviews; focus group
Approach to analysis	Qualitative
Effect of intervention on UTI versus comparator (if applicable)	N/A
Side effects of treatment (if applicable)	N/A
Evidence of adherence to treatment (if applicable)	N/A
Conclusions	Need to change the beliefs of nursing home staff about UTIs and their management to deliver sustainable change in antibiotic prescribing behaviour.
Limitations	Small sample – one nursing home.<br>Authors note that patient perspective may be underrepresented as the two senior citizens who participated as stakeholders were a retired GP and a retired nurse. Nursing home residents were not included. The only nursing home informants in the pilot study were registered nurses (n=2) as other staff were absent (due to holiday or illness). Approach to and use of participatory observations not described in the paper other than stating these were done in 5 nursing homes by the primary investigator.
Companion papers	Protocol for the RCT (Arnold et al, 2020) gives additional background and care home context relating to UTI.

<table>
<tr><th colspan="6">Topic 1: Developing interventions to optimise good practice</th></tr>
<tr><td colspan="6">Prompts:</td></tr>
<tr><td>bundled care</td><td>tools for assessing soft signs, decision-support, structured communication</td><td>Roles, responsibilities and skills of staff</td><td>aligned to existing priorities</td><td>support from GP and other health professionals</td><td>other</td></tr>
<tr><td colspan="6">Actions before contacting the GP added to the tool, including increased observation of the resident and preventative hygienic measures (e.g., improved intimate hygiene). Tool adapted to accommodate a checkbox to record non-specific symptoms, together with a definition of delirium to avoid confusion between the two. Space to record changes in urine (smelly, unclear) added even though the researchers recognised this is not a diagnostic sign as nursing home staff searched for a space to put this and leaving it out could become a barrier to using the dialogue tool. Final intervention contains a dialogue tool (comprising a reflection (to prompt discussion with colleagues and embed UTI learning) and a communication component (to structure clinical history collection, communication of clinical history and decision making around if UTI and treatment required) and a case-based educational session (to address UT knowledge gaps and introduce the dialogue tool).
Healthcare helpers communicate their observations to the healthcare assistant or registered nurse, who are the only ones permitted to contact the GP directly. When the GP is not available to discuss directly with nursing home staff (found to be the most common situation) a message is conveyed to the GP by the medical secretary. Nursing home staff work with a spectrum of non-specific behavioural symptoms ranging from delirium to slight change. They understand that delirium requires immediate medical attention and a slight change requires observation and preventative measures such as ensuring fluid intake. "Sound knowledge of UTIs and good communication skills are central to providing sufficient and correct information for physicians to make an appropriate treatment decision. "Care home staff first noticing/evaluating possible symptoms of UTI in complex residents were the least clinically trained for this. Communication of UTI passed through a number of professionals (healthcare assistants, nurses, GPs, medical secretaries). History taking and reporting unstructured and could focus on known and insignificant details. Not recognising UTI as diagnosis of exclusion, expectation that non-specific symptoms indicated UTI. Reliance on intuitive reasoning leading to suspicion of UTI.</td></tr>
<tr><td colspan="6">How is care relating to UTI prevention and recognition integrated into daily care and prioritised?</td></tr>
<tr><td colspan="6">Healthcare helpers and assistants are the first to notice if a nursing home resident appears different than usual. Healthcare assistants and nurses equated mild confusion with delirium and saw this as a symptom of UTI. Fear of missing a UTI diagnosis appeared to underpin this, which also explained why they found it hard to disregard a positive urine (dipstick) test. All healthcare staff involved in the tailoring of the tool equated a positive urine tests smelly and unclear urine with UTI. They uncritically suspected UTI when they observed a nonspecific symptom. Responsibility for UTI prevention and recognition is with healthcare helpers and healthcare assistants who recognise subtle changes that may indicate UTI.</td></tr>
<tr><td colspan="6">Other findings of relevance (not relating to prompts)</td></tr>
<tr><td colspan="6">Objective, measurable results of urine tests prioritised by GP over subjective assessments of signs and symptoms ('soft signs') when making a diagnosis. Interviews with nursing home staff found that when using the decision flowchart with a clinical case of a resident with non-specific behavioural changes and smelly urine, their intuitive reasoning led to inappropriate suspicion of UTIs even though they recognised urinary symptoms were absent. Nursing assistant said she ignored the other possible paths in the flowchart when the resident had light confusion, seeing this (incorrectly) as "one or more constitutional symptoms" of UTI. Findings revealed it can be difficult for nursing home staff to change their intuitive understandings of what constitutes a UTI. The tool incorporated a reflective discussion section for staff to check their understanding and learn from each other. Education session included content designed to shift thinking towards UTI as a diagnosis of exclusion (ruling out alternative causes of symptoms before deciding on UTI). Researchers explained findings according to the dual process theory of cognition (intuitive and analytical system - thinking fast and slow) and also cognitive dissonance to explain the reaction of staff when their knowledge and practice were misaligned with the definition of UTI used in the reflection tool.</td></tr>
<tr><td colspan="6">Is the evidence provided in this theory area good and relevant enough to be included in the synthesis (consider issues of sample size, data collection, data analysis, claims made and any limitations)?</td></tr>
<tr><td colspan="6">Yes</td></tr>
</table>

<table>
<tr><th colspan="5">Topic 2: Delivering and sustaining good practice</th></tr>
<tr><td colspan="5">Prompts:</td></tr>
<tr><td>multi-faceted design</td><td>skilled facilitation</td><td>tailored mixed mode education, huddles</td><td>monitoring and feedback</td><td>champions as change agents</td></tr>
<tr><td>successful support tools</td><td>management support</td><td>campaigns</td><td>regulatory incentives and oversight</td><td>other</td></tr>
<tr><td colspan="5"></td></tr>
<tr><td colspan="5">How is the intervention delivered and what impact does this have on implementation of practices to recognise and prevent UTI?</td></tr>
<tr><td colspan="5">The plan for the intervention is to introduce the tool through a case-based education session that addressed UTI knowledge gaps and demonstrated the use of the tool. The tool would be used as part of the clinical history gathering and reporting and as reflection within the care home team for what the preventative measures and diagnosis were. The intervention aims to address knowledge gaps, address commonly held misunderstandings of UTI to develop and embed more evidence-based understanding across all care home staff through discussion/reflection with colleagues to check observations, thinking and actions. To improve clinical history gathering and reporting within the care home and when communicating with GPs.</td></tr>
<tr><td colspan="5">Other findings of relevance (not relating to prompts)</td></tr>
<tr><td colspan="5"></td></tr>
<tr><td colspan="5">Is the evidence provided in this theory area good and relevant enough to be included in the synthesis (consider issues of sample size, data collection, data analysis, claims made and any limitations)?</td></tr>
<tr><td colspan="5">No</td></tr>
</table>

<table>
<tr><th colspan="5">Topic 3: Care home context and culture</th></tr>
<tr><td colspan="5">Prompts:</td></tr>
<tr><td>empowered and developed workforce</td><td>effective leadership</td><td>optimised staffing & skill mix</td><td>safety culture embedded</td><td>engaged in audit and QI</td></tr>
<tr><td>UTI recognised as preventable</td><td>external regulation</td><td>involves resident and family</td><td>engaged and empowered residents</td><td>other</td></tr>
<tr><td colspan="5"></td></tr>
<tr><td colspan="5">How do individuals/teams and residents/families become empowered to engage in UTI prevention and recognition?
By recognising the roles each staff member has in recognition of UTI, the intervention was developed to be appropriate to their expected level of clinical / resident knowledge (e.g. working with their knowledge about how a resident is when well, their understanding of the signs of UTI, the language they are familiar with for UTI) The tool allows for structured information gathering and communication with colleagues and GPs.</td></tr>
<tr><td colspan="5">Other findings of relevance (not relating to prompts)</td></tr>
<tr><td colspan="5">Knowledge of UTI - consider the 'reverse triangle' that UTI is a diagnosis of exclusion rather than the default diagnosis.</td></tr>
<tr><td colspan="5">Is the evidence provided in this theory area good and relevant enough to be included in the synthesis (consider issues of sample size, data collection, data analysis, claims made and any limitations)?</td></tr>
<tr><td colspan="5">Yes</td></tr>
</table>

**Topic 4: Co-design and multi-agency working**					
Prompts:					
tailored to context	*common language*	*shared goals*	*aligned to existing priorities*	*support from GP and other healthcare professionals*	*other*
Tool tailored to needs of staff across 5 cycles. This included: including a space to record intuitive and non-evidence based but commonly held beliefs of the signs of UTI (e.g. look and smell of UTI. Space to record dipstick results (as this was an expected test by GPs) Simplifying language. Focus on gathering and communicating clinically relevant information.<br>Adapted Loeb diagnostic algorithm originally developed for physicians and nurses for use by healthcare helpers, assistants and nurses using vocabulary understandable to them.<br>GPs reported that they thought the nursing home staff sometimes contacted general practice too early in the illness.					
What is the nature of relationships between care home staff and the healthcare professionals/agencies they work with and how do relationships impact on involvement in UTI prevention and recognition?					
"The workflow in the nursing homes and the communication pathway from the bedside of the resident to the GP mean that the task of evaluating highly complex patients falls on the healthcare staff<br>least trained for it." "The information about the condition of the residents passes through many actors before reaching the GP." "Nursing home staff<br>'s reports to the GP about the conditions of the residents often included insignificant deviations from the norm, and descriptions were often vague." Paper highlights some issues of trust that GPs have with care home staff. This appears to be through missing information, concerns that some contact is unnecessary and communication that is not focused. Communication for UTI is usually conducted via telephone and GPs rarely visit for UTI, prescribing based on the information they are provided by the care home.					
How does the work of UTI prevention and recognition align with / relate to other priorities?					
Seems to be a disconnect between prevention (not really discussed and preventative or non-ABs measures not considered (according to GPs) and recognition in that many non-specific symptoms considered to be linked to UTI. Shows high awareness of possibility of UTI, concerns around not spotting UTI – but suggests limited understanding of prevention and alternative methods.					
Other findings of relevance (not relating to prompts)					
Protocol for the RCT gives additional background and care home context relating to UTI.					
Is the evidence provided in this theory area good and relevant enough to be included in the synthesis (consider issues of sample size, data collection, data analysis, claims made and any limitations)?					
Yes					
