# Supplementary figures and images for: Preventing urinary tract infection in older people living in care homes: the ‘StOP UTI’ realist synthesis
Source: BMJ Qual Saf. 2024 Aug 8;34(3):e016967. doi: 10.1136/bmjqs-2023-016967 (PMC11874410; doi:10.1136/bmjqs-2023-016967)

Supplementary File 6: Figure 6 Summary of the search process

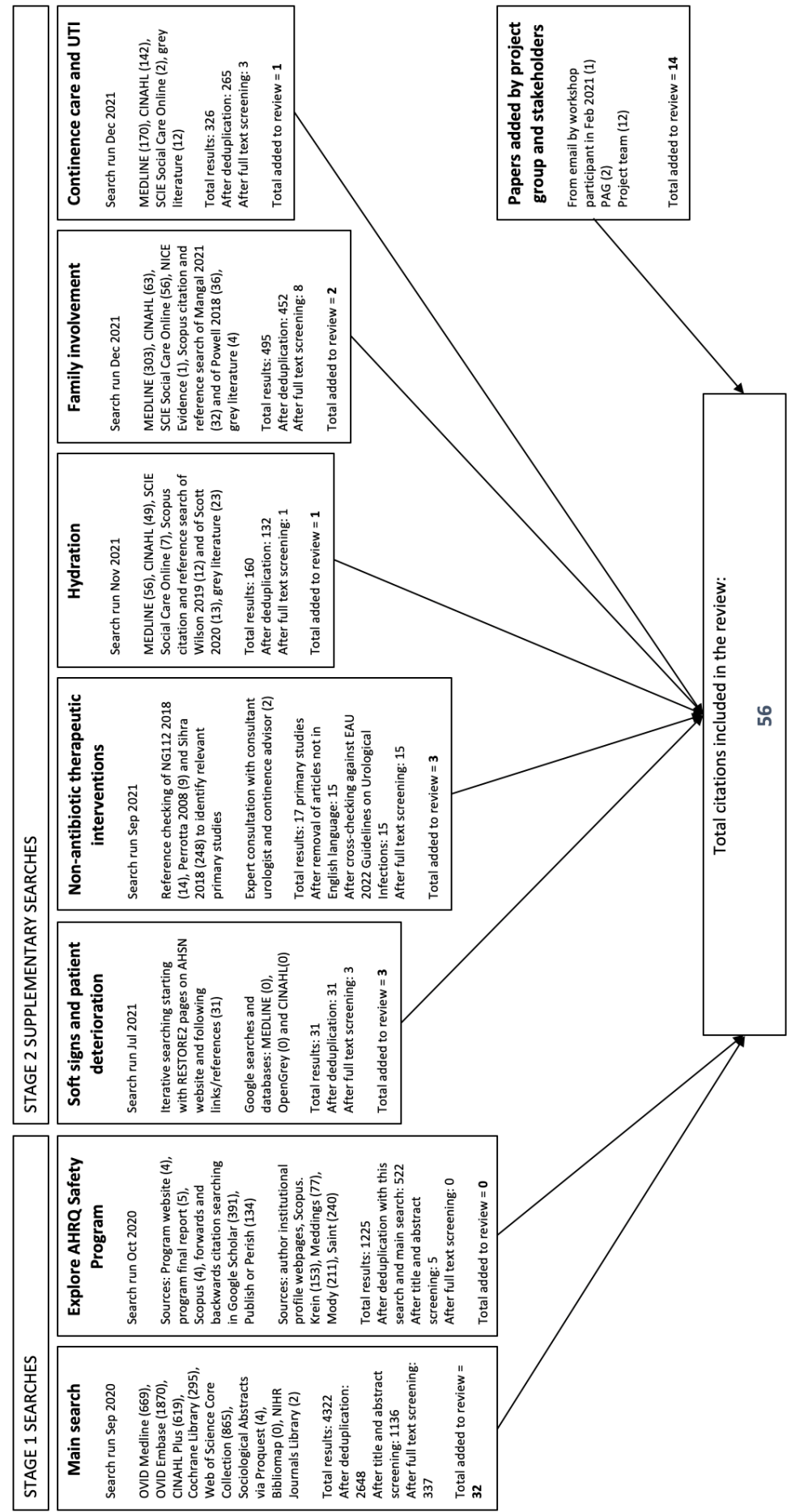

Supplement: online supplemental file 6 [file bmjqs-34-3-s006.pdf]
