# Supplementary material for: Preventing urinary tract infection in older people living in care homes: the ‘StOP UTI’ realist synthesis
Source: BMJ Qual Saf. 2024 Aug 8;34(3):e016967. doi: 10.1136/bmjqs-2023-016967 (PMC11874410; doi:10.1136/bmjqs-2023-016967)
Supplement: online supplemental file 7 [file bmjqs-34-3-s007.pdf]

Supplementary File 7: Table 3 List of included studies

Authors and date	Title	Source	Country	Practice setting	CMOc
Abusalem *et al*. (2019)	The Relationship Between Culture of Safety and Rate of Adverse Events in Long-Term Care Facilities	Journal of Patient Safety 2019; 17(4):299-304.	USA	Long term care facilities	8
Allemann and Sund-Levander (2015)	Nurses' actions in response to nursing assistants' observations of signs and symptoms of infections among nursing home residents	Nursing Open 2015; Nov; 2(3): 97-104.	Sweden	Nursing homes	2
Arnold *et al*. (2021)	Effectiveness of a tailored intervention to reduce antibiotics for urinary tract infections in nursing home residents: a cluster, randomised controlled trial	The Lancet Infectious Diseases 2021; Nov;21(11):1549-1556.	Denmark	Nursing homes	1, 2, 3, 8
Arnold *et al*. (2020)	Development of a Tailored, Complex Intervention for Clinical Reflection and Communication about Suspected Urinary Tract Infections in Nursing Home Residents	Antibiotics 2020; Jun 25; 9(6): 360.	Denmark	Nursing homes	1, 2, 3, 9
Ashraf *et al*. (2020)	Diagnosis, Treatment, and Prevention of Urinary Tract Infections in Post-Acute and Long-Term Care Settings: A Consensus Statement from AMDA's Infection Advisory Subcommittee	The Journal of the American Medical Directors Association 2020;21(1):12-24.	USA	Post-acute and long-term care settings	3
Atkins *et al*. (2020)	Reducing catheter-associated urinary tract infections: a systematic review of barriers and facilitators and strategic behavioural analysis of interventions	Implementation Science 2020; 15:44.	N/A	N/A	6
Bak *et al*. (2018)	Drinking vessel preferences in older nursing home residents: optimal design and potential for increasing fluid intake	British Journal of Nursing 2018; 27(22): 1298-1304.	UK	Care homes	4
Beeber et al. (2021)	Nurse decision-making for suspected urinary tract infections in nursing homes: potential targets to reduce antibiotic overuse.	Journal of the American Medical Directors Association 2021; 22(1): 156-163.	USA	Nursing homes	1, 3
Bonkat *et al*. (2022)	European Association of Urology Guidelines on urological infections	https://uroweb. org/guidelines/ urological- infections	N/A	N/A	7
Booth *et al.* (2019)	Evaluating a hydration intervention (DRInK Up) to prevent urinary tract infection in care home residents: A mixed methods exploratory study	Journal of Frailty, Sarcopenia and Falls 2019; Jun;4(2):36.	UK	Care homes	4, 5
Bunn *et al*. (2018)	Supporting shared decision making for older people with multiple health and social care needs: a realist synthesis	BMC Geriatrics 2018; Dec; 18(1):1-5.	UK	Care homes	3
Burton *et al*. (2021)	Theory and practical guidance on de-implementation of practices across health and care services: a realist synthesis	NIHR Journals Library Health Serv Deliv Res 2021;9(2).	UK	NHS	3

Buswell *et al.* (2017)	What Works to Improve and Manage Faecal Incontinence in Care Home Residents Living with Dementia? A Realist Synthesis of the Evidence (FINCH)	Journal of the American Medical Directors Association 2017; 18:752-760.	UK	Care homes	8
Chaaban *et al.* (2019)	Decisional issues in antibiotic prescribing in French nursing homes: An ethnographic study	Journal of Public Health Research 2019; Nov 4; 8(2):jphr-2019.	France	Nursing homes	2
Chambers *et al.* (2019)	A recipe for antimicrobial stewardship success: Using intervention mapping to develop a program to reduce antibiotic overuse in long-term care	Infection Control and Hospital Epidemiology 2019; Jan; 40(1):24-31.	Canada	Long-term care	1, 3, 7
Dongjuin *et al.* (2019)	Relationship between nursing home quality indicators and potentially preventable hospitalisation	BMJ Quality and Safety 2019; 28(7):524-533.	USA	Nursing homes	8
Downs *et al.* (2021)	A complex intervention to reduce avoidable hospital admissions in nursing homes: a research programme including the BHiRCH-NH pilot cluster RCT 2021	NIHR Journals Library 2021 Feb. Programme Grants for Applied Research doi: 10.3310/pgfar09020.	UK	Nursing homes	2, 8, 9
Fleming *et al.* (2015)	Antibiotic Prescribing in Long-Term Care Facilities: A Meta-synthesis of Qualitative Research	Drugs and Aging 2015; Apr; 32(4):295-303.	N/A	Long term care facilities	3
Greene *et al.* (2018)	I-Hydrate training intervention for staff working in a care home setting: An observational study	Nurse Education Today 2018; Sep 1;68:61-5.	UK	Care Homes	4, 8, 9
Haunch *et al.* (2021)	Understanding the staff behaviours that promote quality for older people living in long term care facilities: A realist review	International Journal of Nursing Studies 2021; May; 117:103905.	UK	Long-term residential care facilities	8
Hughes *et al.* (2020)	A multifaceted intervention to reduce antimicrobial prescribing in care homes: a non-randomised feasibility study and process evaluation	NIHR Journal Library Health Serv Deliv Res 2020; 8(8).	UK	Care homes	1, 2, 3, 8, 9
Jones *et al.* (2020)	Development of an information leaflet and diagnostic flow chart to improve the management of urinary tract infections in older adults: a qualitative study using the Theoretical Domains Framework	BJGP Open 2020; 4(3) doi: 10.3399/bjgpopen20X101044.	UK	Care homes	1, 3, 7
Jones *et al.* (2021)	A Qualitative Investigation of the Acceptability and Feasibility of a Urinary Tract Infection Patient Information Leaflet for Older Adults and Their Carers	Antibiotics 2021; 10(1):83 doi: 10.3390/ antibiotics10010083.	UK	Care homes and primary care	1, 3, 7
Kelley *et al.* (2020)	The influence of care home managers on the implementation of a complex intervention: findings from the process evaluation of a randomised controlled trial of dementia care mapping	BMC Geriatrics 2020; 20:303.	UK	Care homes	8
Klay and Marfyak (2005)	Use of continence nurse specialist in extended care facility	Urologic Nursing 2005; 25:2; ProQuest One Academic. pg. 101	USA	Long term care facilities	7

Kousgaard *et al*. (2022)	Implementing an intervention to reduce use of antibiotics for suspected urinary tract infection in nursing homes – a qualitative study of barriers and enablers based on Normalization Process Theory	BMC Geriatrics 2022; 22:265 doi: 10.1186/ s12877-022-02977-w.	Denmark	Nursing homes	1, 8, 9
Krein *et al*. (2017)	A national collaborative approach to reduce catheter-associated urinary tract infections in nursing homes: A qualitative assessment	American Journal of Infection Control 2017; December 01; 45(12): 1342–1348 doi:10.1016/ j.ajic.2017.07.006.	USA	Nursing homes	6, 8, 9
Lean *et al*. (2019)	Reducing urinary tract infections in care homes by improving hydration	BMJ Open Quality 2019; 8:e000563 doi:10.1136/bmjoq-2018-000563	UK	Care homes	4, 5, 8
Long *et al*. (2013)	Practice brief: A hydration initiative in a long-term care facility	Geriatric Nursing 2013; 34; 339-343.	USA	Long term care	4, 5
Low *et al*. (2015)	A Systematic Review of Interventions to Change Staff Care Practices in Order to Improve Resident Outcomes in Nursing Homes	PLoS ONE 10(11): e0140711 doi:10.1371/ journal.pone.0140711	N/A	Nursing homes	8
Marshall *et al*. (2018)	An evaluation of a safety improvement intervention in care homes in England: a participatory qualitative study	Journal of the Royal Society of Medicine 2018; 111(11):414–421 doi: 10.1177/0141076818803457.	UK	Care homes	8
McNulty *et al*. (2008)	Exploring reasons for variation in urinary catheterisation prevalence in care homes: a qualitative study	Age and Ageing 2008; Nov 1; 37(6):706-10.	UK	Care homes	8
Meddings *et al*. (2017)	Systematic Review of Interventions to Reduce Urinary Tract Infection in Nursing Home Residents	Journal of Hospital Medicine 2017; 12(5):365-8.	N/A	Nursing homes	6
Mody *et al*. (2015)	A targeted infection prevention intervention in nursing home residents with indwelling devices: a randomized clinical trial	Clin Infect Disease 2015; 61(1):86-94.	USA	Nursing homes	9
Mody *et al*. 2017)	A National Implementation Project to Prevent Catheter-Associated Urinary Tract Infection in Nursing Home Residents	JAMA Internal Medicine 2017; 177(8):1154-1162.	USA	Nursing homes	6, 8, 9
Nace *et al*. (2020)	A Multifaceted Antimicrobial Stewardship Program for the Treatment of Uncomplicated Cystitis in Nursing Home Residents	JAMA Intern Med 2020; Jul 1;180(7):944-951.	USA	Nursing homes	3
NICE (2018)	NICE Guideline 112. Urinary tract infection (recurrent): antimicrobial prescribing.	National Institute for Health and Care Excellence 2018.	England, UK	N/A	7
Ouslander *et al*. (2011)	Interventions to Reduce Hospitalizations from Nursing Homes: Evaluation of the INTERACT II Collaborative Quality Improvement Project	Journal of the American Geriatrics Society 2011; 59(4):745-753.	USA	Nursing homes	8

Pasay *et al*. (2019)	Antimicrobial stewardship in rural nursing homes: Impact of interprofessional education and clinical decision tool implementation on urinary tract infection treatment in a cluster randomized trial	Infection Control and Hospital Epidemiology 2019; Apr; 40(4):432-7.	Canada	Nursing homes	1, 2, 3
Pettersson *et al*. (2011)	Can a multifaceted educational intervention targeting both nurses and physicians change the prescribing of antibiotics to nursing home residents? A cluster randomized controlled trial	Journal of Antimicrobial Chemotherapy 2011; Nov 1;66(11):2659-66.	Sweden	Nursing homes	3
Potter *et al*. (2019)	Multifaceted intervention to Reduce Antimicrobial Prescribing in Care Homes: a process evaluation of a UK-based non-randomised feasibility study (REACH study)	BMJ Open 2019; Nov 1; 9(11): e032185.	UK	Care homes (nursing and residential)	3, 8
Powell *et al*. (2018)	Family involvement in timely detection of changes in health of nursing homes residents: a qualitative exploratory study	Journal of Clinical Nursing 2018; Jan;27(1-2):317-27.	UK	Nursing homes	3
Rycroft-Malone *et al*. (2018)	A realist process evaluation within the Facilitating Implementation of Research Evidence (FIRE) cluster randomised controlled international trial: an exemplar	Implementation Science 2018; 13:138.	England, Netherlands, Republic of Ireland and Sweden	Care homes	8
Scanlon *et al*. (2012)	Reducing catheter-associated urinary tract infections in home care: a performance improvement project	Nurs Econ 2017; 35(3):134-141.	USA	Home care	8
Shallcross *et al*. (2021)	Antibiotic prescribing for lower UTI in elderly patients in primary care and risk of bloodstream infection: A cohort study using electronic health records in England	PLOS Medicine 2020; Sep 21; 17(9): e1003336.	England, UK	Adults aged 65 years and older in primary care	3
Sihra *et al*. (2018)	Nonantibiotic prevention and management of recurrent urinary tract infection	Nature Reviews Urology 2018; Oct 25:1.	N/A	N/A	7
Smith *et al*. (2018)	Evaluation of the association between Nursing Home Survey on Patient Safety culture (NHSOPS) measures and catheter-associated urinary tract infections: results of a national collaborative	BMJ Quality and Safety 2018; Jun 1; 27(6):464-73.	USA	Nursing homes	8
Sreedhara *et al*. (2015)	Nursing home leaders' views on catheter use and CAUTI as a quality and safety concern	Journal of The American Geriatrics Society 2015; Apr 1; (63):S213-S214.	USA	Nursing homes	8
Sund-Levander and Tingstrom (2013)	Clinical decision-making process for early nonspecific signs of infection in institutionalised elderly persons: experience of nursing assistants	Scandinavian Journal of Caring Sciences 2013; Mar;27(1):27-35.	Sweden	Nursing homes and home care	1, 2
Surr *et al*. (2019)	Exploring the role of external experts in supporting staff to implement psychosocial interventions in care home settings: results from the process evaluation of a randomized controlled trial	BMC Health Services Research 2019; 19:790.	UK	Care homes	8, 9

Tingstrom *et al*. (2010)	Early nonspecific signs and symptoms of infection in institutionalized elderly persons: perceptions of nursing assistants	Scandinavian Journal of Caring Sciences 2010; 24:24-31.	Sweden	Nursing homes	1, 2
van Buul *et al*. (2014)	Factors influencing antibiotic prescribing in long-term care facilities: a qualitative in-depth study	BMC Geriatrics 2014; Dec;14(1):1-1.	Netherlands	Nursing homes	2
Williams *et al*. (2016)	Improving skills and care standards in the support workforce for older people: a realist synthesis of workforce development interventions	BMJ Open 2016; 6:e011964.	UK	Health and social care services for older people	9
Wilson *et al*. (2019)	Improving hydration of care home residents by increasing choice and opportunity to drink: A quality improvement study	Clinical Nutrition 2019; 38:1820-1827.	UK	Care homes	4, 5, 8
Wilson *et al*. (2020)	Improving fluid consumption of older people in care homes: an exploration of the factors contributing to under-hydration	Nursing and Residential Care 2020; 22(3) doi:10.12968/nrec.2020.22.3.139.	UK	Care homes	4, 5, 9
Wanat *et al*. (2020)	Optimising Interventions for Catheter-Associated Urinary Tract Infections (CAUTI) in Primary, Secondary and Care Home Settings	Antibiotics 2020; 9:419 doi:10.3390/antibiotics9070419.	USA	Primary, secondary and care home settings	6
Xu *et al*. (2019)	Relationship between nursing home quality indicators and potentially preventable hospitalisation	BMJ Quality and Safety 2019; 28(7):524-533.	USA	Nursing homes	8
