# Supplementary material for: Preventing urinary tract infection in older people living in care homes: the ‘StOP UTI’ realist synthesis
Source: BMJ Qual Saf. 2024 Aug 8;34(3):e016967. doi: 10.1136/bmjqs-2023-016967 (PMC11874410; doi:10.1136/bmjqs-2023-016967)
Supplement: online supplemental file 8 [file bmjqs-34-3-s008.pdf]

Supplementary File 8: Table 4 Summary of context-mechanism-outcome configurations (CMOc)

Theory area 1: Strategies to support accurate recognition of UTI		
CONTEXT	MECHANISM	OUTCOME
**CMOc 1: Recognition of UTI is informed by skills in clinical reasoning**		
Care staff receive education on UTI and its diagnosis that addresses their beliefs and misconceptions<br><br>Education is tailored to the role and work of care staff, supporting development of clinical reasoning skills through application to practical examples<br><br>Regular opportunities to embed learning are taken through discussion and reflection on whether changes in a resident's condition are due to UTI<br><br>Application of learning to practice is facilitated by managers and senior care staff	**Resource**<br>Care staff are enabled to develop an accurate understanding of what constitutes a UTI and are supported to interpret signs and symptoms<br><br>**Reasoning/response**<br>Care staff have the knowledge and confidence to consider alternative explanations for a change in a resident's condition<br><br>Care staff do not rely on non-evidence-based signs and symptoms (e.g., changes to the colour and smell of urine) or use of urine dipsticks to decide if a resident may have a UTI	Reduced likelihood of UTI being the default explanation for generalised changes in a resident<br><br>Reduced reliance on urine dipsticks<br><br>Increased likelihood that the resident's care will be managed appropriately<br><br>Reduction in inappropriate prescribing of antimicrobial agents
**CMOc 2: Decision support tools enable a whole care team approach to communication**		
Structured decision support tools for diagnosing UTI are co-designed by the care team to fit with existing processes and reflect symptom presentations seen by care staff<br><br>The whole care team are actively involved in recognition and prevention of UTI and see the relevance to their role<br><br>Regular opportunities to apply decision support tools are taken to discuss and reflect upon their value in more accurately identifying signs and symptoms of infection	**Resource**<br>Care staff are enabled to use a shared language to convey accurate and relevant information about a resident's signs and symptoms<br><br>**Reasoning/response**<br>Care staff see value in the use of structured tools, together with their knowledge of a resident, to support the accurate diagnosis of UTI<br><br>Health care support workers feel motivated to communicate their observations and believe their concerns will be listened to and acted upon<br><br>Care staff have more confidence in evaluating early 'soft signs' and suspicions of UTI and determining when to escalate their concerns to the general practitioner (GP)	General Practitioner more likely to regard staff concerns as valid<br><br>Diagnoses of UTI more likely to be accurate<br><br>Reduction in inappropriate prescribing of antimicrobial agents
**CMOc 3: Active monitoring is recognised as a legitimate care routine for UTI**		
The care team and family carers accept active monitoring as a proactive step when there is diagnostic uncertainty about non-specific signs and symptoms of UTI<br><br>A structured approach to active monitoring is supported by a protocol with clearly defined actions and criteria for escalation	**Resource**<br>The care team are empowered to engage in active monitoring and evaluate causes of non-specific signs and symptoms<br><br>**Reasoning/response**	Reduced reliance on low value diagnostic practices and non-evidence-based decision-making<br><br>Reduced risk of incorrect UTI diagnosis, missing a

Residents and family carers are engaged in active monitoring and included in discussions of the benefits and risks of different actions	Concerns of the resident, family carers and staff are respected, and their expectations managed through their involvement in shared decision-making<br><br>Effective communication occurs between care home staff, residents and family carers and the primary care team<br><br>Care staff understand the actions they should take and when to escalate concerns about a resident's condition	UTI or other infection or condition<br><br>Reduction in inappropriate prescribing of antimicrobial agents
**Theory area 2: Care strategies for residents to prevent UTI and catheter-associated UTI (CAUTI)**		
**CMOc 4: Hydration is recognised as a care priority for all residents**		
Hydration is prioritised as a core activity by managers and others involved in planning care<br><br>The care home provides the resources required to support hydration to meet residents' needs and preferences<br><br>Care staff receive effective* education and training on the risk of dehydration in the elderly and the importance of hydration in preventing UTI	**Resource**<br>Care routines incorporate and prioritise sufficient opportunities for residents to drink<br><br>Adequate resources to support residents to drink are available e.g., wide range of fluids, drink stations, suitable drinking vessels, systems for distributing drinks<br><br>**Reasoning/response**<br>Staff understand the importance of hydration, recognise it as a priority and are empowered to embed support for residents to drink as a core activity in their work routines	Residents consume sufficient fluids every day<br><br>Number of UTI are reduced
**CMOc 5: Systems are in place to drive action that helps residents to drink more**		
General or individual targets for residents' fluid intake are informed by staff who care for them and are supported by the care home<br><br>Systems are in place to measure daily fluid intake accurately and alert care staff to residents whose fluid consumption is poor<br><br>Residents and their families are involved in setting targets and supporting fluid consumption	**Resource**<br>Care staff set realistic target fluid intakes, which are routinely monitored and reviewed<br><br>Actions are agreed to manage a resident whose fluid intake is inadequate<br><br>**Reasoning/response**<br>Care home staff are enabled and motivated to respond to targets, record actual intake and take corrective action to increase the resident's fluid intake	Residents with poor fluid intake are identified early and actions implemented in a timely way to reduce the risk of UTI
**CMOc 6: Good infection prevention practice is applied to indwelling urinary catheters**		
Care home managers recognise the benefit of CAUTI prevention and improvement strategies and are supported by expert practitioners and regulators<br><br>Staff education and training* develops positive attitudes towards minimising the use of catheters, applying infection prevention practice to their management and recognising signs and symptoms of CAUTI<br><br>Tools are available to assist care home staff in assessing the need for an indwelling urinary catheter and prompt removal<br><br>Residents and family carers are involved in decision about urinary catheters and IP practice related to their care	**Resource**<br>Care staff recognise CAUTI as a significant health problem and understand their role in its prevention and diagnosis<br><br>**Reasoning/response**<br>Care staff are less accepting of the use of indwelling urinary catheters and are enabled to challenge their use and initiate removal<br><br>Care staff have confidence to apply infection prevention practice to the management of indwelling urinary catheters<br><br>Care staff understand the signs and symptoms of CAUTI and are less likely to mis-identify CAUTI	Reduction in catheter days and CAUTI<br><br>Reduction in inappropriate prescribing of antimicrobial agents

**CMOc 7: Proactive Strategies are in place to prevent recurrent UTI (RUTI)**		
Care staff recognise recurrent UTI as a health problem and driver for antimicrobial resistance, and that potentially effective preventable treatment options are available<br><br>Specialist input to identify appropriate treatment options for residents with recurrent UTI is available through the GP, community pharmacists or referral to local continence advisor or urology service<br><br>Systems that alert care home staff and primary care practitioners to residents at risk of recurrent UTI	**Resource**<br>Residents with recurrent UTI are identified<br><br>Proactive management, including non-antimicrobial pharmacological interventions, is enabled to reduce the risk of reoccurrence<br><br>**Reasoning/response**<br>Care staff are less accepting of the inevitability of recurrent UTI and consider a wider range of options to prevent reoccurrence	Residents with recurrent UTI are offered preventative treatment<br><br>Reduction in number of UTI<br><br>Risk of UTI caused by antimicrobial resistant pathogens is reduced
**Theory area 3: Making best practice happen**		
**CMOc 8: Care home leadership and culture fosters safe fundamental care**		
Care home managers actively and visibly endorse approaches to care that support a resident-centred culture and improve the quality of life of residents<br><br>Care home managers provide the resources and support needed for their staff to actively engage in implementing and sustaining improvements<br><br>Leadership at the care home is stable<br><br>Improvement initiatives align with the priorities of the care home and assist in meeting quality, regulatory and commissioning requirements	**Resource**<br>Care staff are enabled to commit time to preventative fundamental care, including UTI/CAUTI prevention and recognition<br><br>Unit leaders facilitate collective engagement in improving care, incorporating regular reviews and adaptations to work processes to embed changes into care routines<br><br>Care homes have access to expertise and resources to facilitate improvement and measure change<br><br>**Reasoning/response**<br>Care staff understand what is expected of them and have the confidence to prioritise relevant care activity and work as a team to incorporate changes in work routines<br><br>Care staff recognise the value of activities that prevent UTI/CAUTI and can demonstrate the benefits to residents	There is a continual review of practices for the prevention and recognition of UTI/CAUTI in residents, identifying areas for improvement and seeking ways to sustain best practice<br><br>Care staff have improved job satisfaction for the standard of care they provide
**CMOc 9: Developing knowledgeable care teams through contextualised learning**		
Educational resources align with workforce capabilities, experience and preferred methods of learning<br><br>Education is contextualised to the roles of care staff with different learning needs and is relevant to their practice<br><br>Education supports care staff to develop skills in reflection, leadership, empowering others and creating a safety culture<br><br>Education is informed by specialists	**Resource**<br>Interactive education promotes active learning about how UTI/CAUTI is recognised and prevented to develop new knowledge and skills and challenge pre-existing beliefs<br><br>Practical resources are available at the point of care to remind staff about what they have learned<br><br>**Reasoning/response**<br>Care staff can see the relevance of new learning about UTI and can apply this to the care of residents<br><br>Care staff are engaged and motivated to reflect more critically on their practice and role in improving care	Best practice to prevent and recognise UTI/ CAUTI will be applied to the care of residents

**Refer to CMOc 9 on well-designed education*
