# Supplementary material for: Preventing urinary tract infection in older people living in care homes: the ‘StOP UTI’ realist synthesis
Source: BMJ Qual Saf. 2024 Aug 8;34(3):e016967. doi: 10.1136/bmjqs-2023-016967 (PMC11874410; doi:10.1136/bmjqs-2023-016967)
Supplement: online supplemental file 9 [file bmjqs-34-3-s009.pdf]

Supplementary File 9: Table 5 Key recommendations for practice

CARE HOME PROVIDERS	
**STRATEGIES TO SUPPORT ACCURATE RECOGNITION OF UTI**	
Developing knowledge and skills	• Care staff receive education about UTI that enables them to accurately recognise signs and symptoms and consider alternative explanations for changes in a resident's condition<br>• Senior care staff create regular opportunities for the care team to review residents with a suspected UTI to embed learning though discussion about what is normal for them and whether changes may be due to UTI
Using decision-support tools	• Structured decision support tools are co-designed by the care team to fit with existing processes and reflect symptom presentations seen by care staff<br>• The whole care team sees the relevance of their role in the active recognition of UTI and are involved in discussion and reflection on the application of the decision support tool to accurately recognise UTI
Active monitoring	• A structured approach to 'active monitoring' is supported by a protocol with clearly defined actions and criteria for escalation to a clinician<br>• The involvement of family carers in identifying subtle changes in a resident's condition needs to be supported and given legitimacy and structure within the care home
**CARE STRATEGIES FOR RESIDENTS TO PREVENT UTI/CAUTI**	
Supporting hydration	• Care staff receive education on the importance of hydration and supporting all residents to drink as a care priority to improve health and minimise the risk of UTI and other avoidable conditions<br>• Care routines are designed to incorporate and prioritise sufficient opportunities to support residents to drink the recommended intake every day<br>• Systems are in place to ensure that residents are offered a wide choice of fluids throughout the day in well-designed cups/mugs
Helping residents to drink more	• Care staff set realistic daily fluid target intakes for residents, which are monitored and reviewed regularly<br>• Systems are in place to accurately measure fluid intake, alert staff when a resident's intake is poor and drive action to help the resident drink more<br>• Care home managers provide organisational support and positive reinforcement for hydration as a care priority
Preventing catheter-associated UTI (CAUTI)	• Care staff receive education and training that enables them to recognise CAUTI as an important health problem and apply principles of infection prevention to the care of urinary catheters and recognition of CAUTI<br>• Care home managers understand the benefit of CAUTI prevention and are supported by regulators, together with specialists in infection prevention and control and quality improvement, to implement improvement strategies

	• Tools are available to assist care staff in assessing the need for a catheter and initiating its removal; care staff involve the resident and family carer in these decisions
Preventing recurrent UTI	• Care home staff and primary care practitioners have systems in place to identify residents who experience 3 or more UTI in 12 months<br>• Proactive management is initiated for residents with recurrent UTI, including a personalised multidisciplinary assessment and advice from continence advisory or urology services if necessary
**MAKING BEST PRACTICE HAPPEN**	
Care home leadership	• Care home managers demonstrate their active and visible endorsement for implementing changes in practice that support resident centred care<br>• Staff are given time, support and resources to implement change<br>• Managers and leaders facilitate engagement of the whole care team in improving care and reviewing and adapting work processes to meet improvement aims<br>• Commissioners and regulators of care identify UTI prevention and accurate recognition as a priority area of care for care homes and facilitate access to relevant expertise to support improvement activity
Developing knowledgeable care teams	• Education is effective when it is:<br>o designed to support care staff to develop skills in reflection, creating a safety culture, leadership and empowering others<br>o contextualised to the roles of care staff at different levels and is relevant to their practice<br>o flexible and uses a range of delivery modes<br>o informed by experts<br>• Practical resources at the point of care remind staff about what they have learnt from formal education
**COMMISSIONERS AND REGULATORS**	
Creating a system-wide approach	• Commissioners and regulators of care identify UTI prevention and accurate recognition as a priority area of care for care homes and facilitate access to relevant expertise to support improvement activity<br>• Action is taken to facilitate access to relevant expertise to support multi-disciplinary assessment and treatment plans for residents with recurrent UTI who have the greatest potential to benefit from effective treatment<br>• Work is undertaken to harmonise decision and communication tools for the recognition and prevention of UTI with those focused on recognising deterioration to facilitate their adoption and integration in care homes
